# Serum metabolic fingerprinting of psoriasis and psoriatic arthritis patients using solid-phase microextraction—liquid chromatography—high-resolution mass spectrometry

**DOI:** 10.1007/s11306-021-01805-3

**Published:** 2021-06-16

**Authors:** Nikita Looby, Anna Roszkowska, Nathaly Reyes-Garcés, Miao Yu, Tomasz Bączek, Vathany Kulasingam, Janusz Pawliszyn, Vinod Chandran

**Affiliations:** 1 Department of Chemistry, University of Waterloo, 200 University Avenue, Waterloo, ON N2L 3G1 Canada; 2 Department of Pharmaceutical Chemistry, Medical University of Gdańsk, Gdańsk, Poland; 3 Department of Laboratory Medicine and Pathobiology, University of Toronto, Toronto, Canada; 4 Division of Clinical Biochemistry, University Health Network, Toronto, Canada; 5 Department of Medicine, Division of Rheumatology, University of Toronto, Toronto, Canada; 6 Institute of Medical Science, University of Toronto, Toronto, Canada; 7 Schroeder Arthritis Institute, Krembil Research Institute, University Healthy Network, Toronto, ON MT5 2S8 Canada

**Keywords:** Fatty acids, Psoriasis, Psoriatic arthritis, Serum, Solid-phase microextraction

## Abstract

**Introduction:**

Psoriatic arthritis (PsA), an inflammatory arthritis that develops in individuals with psoriasis, is associated with reduced quality of life. Identifying biomarkers associated with development of PsA as well as with PsA disease activity may help management of psoriatic disease.

**Objectives:**

To use metabolomic fingerprinting to determine potential candidate markers of disease conversion (psoriasis to PsA) and/or PsA activity.

**Methods:**

A novel sample preparation protocol based on solid-phase microextraction (SPME) was used to prepare serum samples obtained from: (1) individuals with psoriasis, some of whom develop psoriatic arthritis (n = 20); (2) individuals with varying PsA activity (mild, moderate, severe; n = 10 each) and (3) healthy controls (n = 10). Metabolomic fingerprinting of the obtained extracts was performed using reversed-phase liquid chromatography coupled to high resolution mass spectrometry.

**Results:**

Psoriasis patients who developed PsA had similar metabolomic profiles to patients with mild PsA and were also indistinguishable from patients with psoriasis who did not develop PsA. Elevated levels of selected long-chain fatty acids (e.g., 3-hydroxytetradecanedioic acid) that are associated with dysregulation of fatty acid metabolism, were observed in patients with severe PsA. In addition, 1,11-undecanedicarboxylic acid—an unusual fatty acid associated with peroxisomal disorders—was also identified as a classifier in PsA patients vs. healthy individuals. Furthermore, a number of different eicosanoids with either pro- or anti-inflammatory properties were detected solely in serum samples of patients with moderate and severe PsA.

**Conclusion:**

A global metabolomics approach was employed to analyze the serum metabolome of patients with psoriasis, PsA, and healthy controls in order to examine potential differences in the biochemical profiles at a metabolite level. A closer examination of circulating metabolites may potentially provide markers of PsA activity.

**Supplementary Information:**

The online version contains supplementary material available at 10.1007/s11306-021-01805-3.

## Introduction

Approximately 3% of North Americans are affected by psoriasis; a chronic, immune-mediated, inflammatory skin disease (Petronic-Rosic & Basko-Plluska, 2012; Rachakonda et al., 2014). While there are many variants of psoriasis—for example, psoriasis vulgaris, guttate psoriasis, palmoplantar pustulosis, and pustular psoriasis—psoriasis vulgaris is the most common, accounting for 85–90% of psoriasis-related conditions (Lowes et al., 2007). Approximately 25% of psoriasis patients also suffer from psoriatic arthritis (PsA), which is a specific form of inflammatory arthritis that may affect peripheral and axial joints and periarticular structures, such as the entheses (Alinaghi et al., 2019). This is a significant problem, as psoriasis and PsA significantly reduce quality of life and often lead to disability and increased mortality (Gladman et al., 2005). Thus, it is important to diagnose PsA as early as possible, as doing so can result in better long-term health outcomes (Haroon et al., 2015). Since PsA often develops after the onset of cutaneous psoriasis, recent research has focussed on converters—psoriatic patients who develop PsA—with the aim of identifying the mechanisms of arthritis development, developing early diagnosis methods, and implementing measures for impeding the disease’s progression, as advances in these areas have the potential to improve patient care tremendously (Abji et al., 2016; Eder et al., 2016, 2017). However, progress towards these goals will require identifying more quantitative biomarkers associated with psoriatic disease pathophysiology and a deeper understanding of the biochemical relevance of these markers in driving disease progression (Petronic-Rosic & Basko-Plluska, 2012).

Researchers have employed several types of targeted and untargeted analyses, otherwise known as ‘omic-related’ approaches, including transcriptomics, proteomics, and genomics among others, to investigate psoriasis development and progression (Jiang et al., 2015). Recently metabolomics—which entails the comprehensive investigation of low molecular weight compounds (metabolites) in a biological system—has provided a new approach that enables disease-related changes to be monitored with greater rapidity than alterations observed at the genome or proteome level (Jiang et al., 2015). Many prior psoriatic-related metabolomics studies have examined mainly the differences between psoriatic patients and healthy individuals. Fewer studies have investigated PsA in particular; these have focused either on how PsA differed from patients with psoriasis (Armstrong et al., 2014); how PsA differed from patients suffering from rheumatoid arthritis (Yan, 2017); or the correlation of PsA with various parameters of disease activity (Coras et al., 2019). Through the analyses of various sample types, including skin, blood components (serum and plasma), and urine, the results of these metabolomics studies yielded biomarkers such as amino acids and various lipids which were associated mainly with psoriasis/PsA severity or how PsA differed from other forms of arthritis (Armstrong et al., 2014; Coras et al., 2019; Kamleh et al., 2015; Li et al., 2017; Ottas et al., 2017; Yan, 2017; Yan et al., 2017). Given the dearth of research related specifically to PsA, the research presented herein attempts to fill that gap by investigating not only psoriatic disease, but also variations in PsA activity explicitly in order to determine how it relates to psoriasis, and if PsA activity pathophysiology may provide insight to disease conversion, potentially indicating a means of prevention or ways of engineering tailored treatments.

In general, metabolomics studies employ either nuclear magnetic resonance (NMR) or high-resolution mass spectrometry (HRMS), both of which are powerful detectors that scan the broad range of metabolites within a given biological sample (Kohler et al., 2016). Regardless of which platform is used, sample preparation remains one of the most critical steps in the metabolomics workflow, as sample pre-treatment can inadvertently affect the outcome of any study. Many HRMS-based metabolomics studies employ standard sample preparation techniques such as protein precipitation (PPt), liquid–liquid extraction (LLE), and solid-phase extraction (SPE) (Gika & Theodoridis, 2011). While these methods are effective, they are also labour intensive, as they require multiple steps in order to obtain the final extract for analysis. Furthermore, the considerable amount of sample handling involved in these preparative procedures can easily lead to errors associated with artefact formation (Vuckovic, 2012). The development of more rapid technologies could both mitigate these issues and increase sample throughput, as the sample would be idle for much less time. Thin-film solid-phase microextraction (TF-SPME) in combination with liquid chromatography (LC) HRMS is one such comparatively faster sample preparation technique that has been reported in numerous metabolomics-related studies (Bojko et al., 2014; Reyes-Garcés et al., 2018; Souza-Silva et al., 2015; Vuckovic & Pawliszyn, 2011). Solid phase microextraction (SPME) is an emerging form of sample preparation that boasts a greener approach to metabolite extraction as a solvent-less technique. Since extraction/isolation of given metabolites from any complex matrices is based on the physiochemical interaction of the metabolites with the polymeric extractant phase immobilized on the SPME device, the sample preparation workflow is simplified. Unlike other popular conventional methods like LLE or PPt which require multiple volumetric extraction steps (based on solvent addition) and usually also require additional steps such as centrifugation and/or sample blowdown, an SPME device can be directly inserted into a complex sample, left for a predetermined amount of time in this sample wherein extraction takes place, followed by a brief rinsing step, and desorption shortly thereafter directly into LC–MS compatible solvents, thereby offering significant sample cleanup and a less encumbered workflow. The SPME process can be fully automated once the samples have been aliquoted, reducing human error and sample handling. Therefore, this work employs TF-SPME coupled to LC-HRMS as a novel and comparatively rapid sample preparation tool for the investigation of PsA pathophysiology by using it to perform metabolomics analyses on serum obtained from three subject groups: patients with varying PsA activity; patients with psoriasis without PsA (PsC), some of whom later develop PsA; and healthy volunteers.

*The objectives of this study were to use TF-SPME-LC-HRMS to find markers or metabolites indicative of*:

1. Conversion from psoriasis to PsA;
2. PsA disease activity.

To the best of our knowledge, this is the first psoriasis-related metabolomics study to investigate the pathology of PsA disease, and further to incorporate the use SPME coupled to LC-HRMS to perform fingerprinting.

## Materials and methods

### Patients

**Fig. 1:**
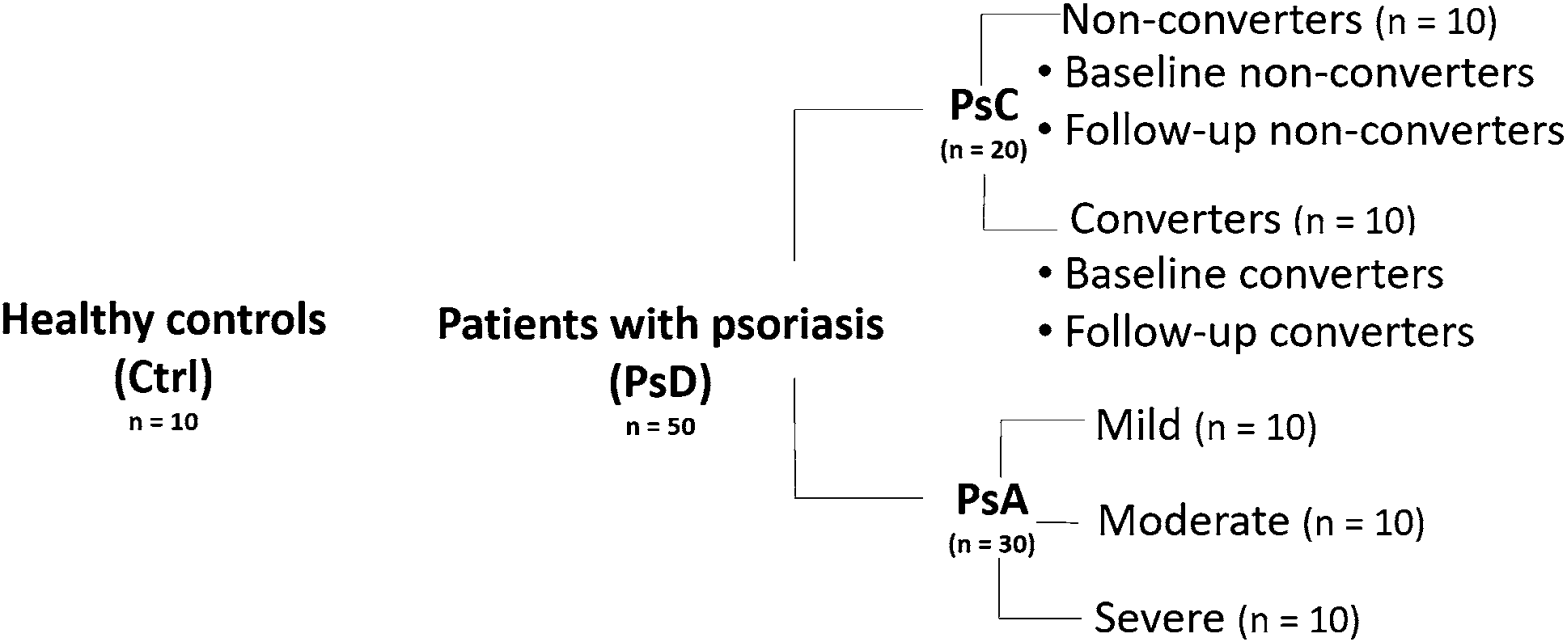
Scheme of patient categories and subcategories of samples collected for analysis. Serum samples were obtained from healthy volunteers serving as controls (Ctrl) and patients with psoriasis (PsD). Patients with psoriasis who may or may not have developed psoriatic arthritis are grouped as converters (PsC); psoriatic patients who have developed psoriatic arthritis are classified as converters, while psoriatic patients who have not developed psoriatic arthritis are classified as non-converters. Samples were collected at baseline—which is prior to development of PsA—from both converters (termed baseline converters) and non-converters (termed baseline non-converters), and at a subsequent follow-up time—after the development of PsA—from both converters (termed follow-up converters) and non-converters (termed follow-up non-converters). Patients with psoriatic arthritis (PsA) were categorized based on their arthritis activity as mild, moderate, or severe. From each PsA group, n = 10 patients were sampled

Serum samples were obtained from the University of Toronto Psoriatic Disease (PsD) Program biobank. Prospective patients with PsA and PsC were enrolled in the program based on careful phenotyping (Gladman & Chandran, 2011), with samples being collected from both types of patients in the program as well as healthy controls without PsD. Patients were assessed using a standard protocol and 68 joints were assessed for tenderness and 66 for swelling by a rheumatologist as recommended by the Group for Research and Assessment of Psoriasis and Psoriatic Arthritis (Duarte-García et al., 2019). These assessments have been previously demonstrated to be reliable (Chandran et al., 2009). A scheme outlining the structure of the patient groups is shown in Fig. 1. The patients and samples obtained are as defined below:

- Ctrl (n = 10): healthy controls
- PsC (n = 20): patients who at study entry were diagnosed with psoriasis by a dermatologist and were evaluated by a rheumatologist to rule out psoriatic arthritis
  - Converters (n = 10): patients with psoriasis who develop psoriatic arthritis
    i. Baseline converters: samples were obtained from psoriasis patients prior to PsA development (conversion)
    ii. Follow-up converters: samples obtained from psoriasis patients post PsA conversion
  - Non-converters (n = 10): patients with psoriasis who do not develop PsA
    i. Baseline non-converters: samples obtained from psoriasis patients
    ii. Follow-up non-converters: samples obtained from psoriasis patients with persisting psoriasis
- PsA (n = 30): Patients with psoriatic arthritis with arthritis activity classified as
- Mild (n = 10):
- Moderate (n = 10):
- Severe (n = 10):

For the PsA subgroup, PsA patient disease activity was arbitrarily classified based on the number of actively inflamed (i.e., swollen or tender) joints, with cases being categorized as either mild (< 4 actively inflamed and 0 swollen joints; n = 10), moderate (4–5 actively inflamed and < 3 swollen joints; n = 10), or severe (> 5 actively inflamed and > 3 swollen joints; n = 10). It is important to note here, that these patients in the PsA subgroup (n = 30) are in fact independent and that each group within this PsA subgroup contains an independent set of individuals who are also different from the patients in the PsC subgroup. More detailed patient information such as sex, age, duration of psoriasis, duration of PsA, treatment, and associated comorbidities is provided in Online Resource 1—Table 1.

### Materials, sample preparation methods, instrumental and data analysis

For more detailed information on sample collection, the materials used in this study, preparation of novel SPME sampling devices, the sample preparation method, instrumental analysis, data pre-treatment and statistical analysis methods, please refer to Online Resource 2.

## Results

**Fig. 2:**
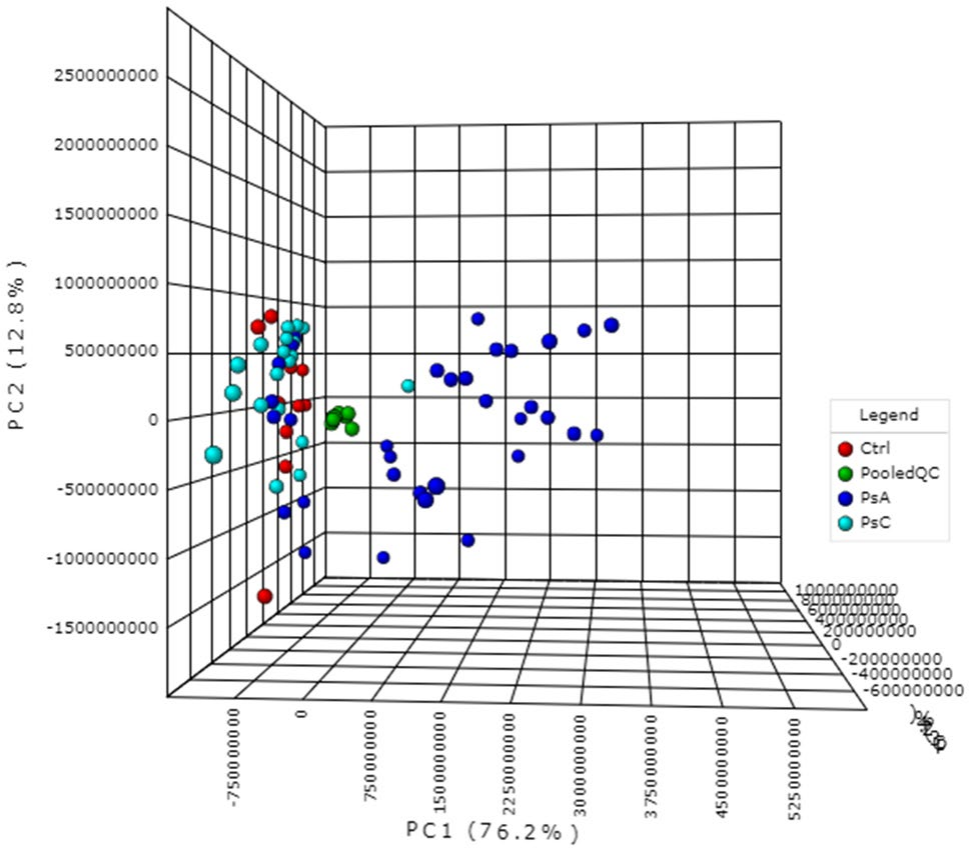
Principal component analysis (PCA–PC1:76.2%, PC2: 12.8%, PC3:2.6%) of the pooled QCs and the three patient groups under investigation: healthy volunteers (Ctrl); patients with varying degrees of psoriatic arthritis (PsA) activity; and patients with psoriasis, but without psoriatic arthritis (PsC), at baseline. The pooled QCs, Ctrls, PsA patients, and PsC patients are represented by green, red, dark blue, and turquoise, respectively. Data shown were obtained via negative mode acquisition and were similar to the results obtained via positive mode acquisition (see Online Resource 1 Fig. 1—pooled QC’s removed)

Metaboanalyst was used to generate a visual representation of the overall structure of all the samples as determined by principal component analysis (PCA) [Fig. 2; Online resource 1—Fig. 1 (positive mode acquisition)]. This unsupervised multivariate approach is ideal for identifying patterns within the data set from which subsequent supervised models may be constructed. PCA with univariate analysis was largely employed for this study as there were only n = 10 samples per group and very close inter-group association (overlap of clusters) among a number of groups, which may result in overfitting and likely failure during the cross-validation of supervised models. However, data from successfully validated models were used for further analyses. PCA was used initially to identify any patient-type differentiation amongst patients classified as PsC (baseline converters and baseline non-converters; n = 20), patients with PsA (mild, moderate and severe, n = 30), and the healthy controls (Ctrls; n = 10). The quality of the resulting data was also assessed via a PCA plot investigating the spread and location of the pooled QC samples on the plot (shown in green in Fig. 2). Since the pooled QCs are a pool of each sample, and were injected every 10 samples throughout instrumental acquisition, their very tight clustering in the middle of the plot strongly indicates stability during instrumental acquisition. Finally, in accordance with this project’s objectives, further investigations were conducted with respect to:

1. The structure of the PsC group—in order to examine initially the baseline metabolic differences between patients with psoriasis who developed PsA (baseline converters) vs. patients with psoriasis who did not (baseline non-converters);
2. The relationship between PsC and PsA—to study indications or markers of PsA disease activity in newly converted patients vs. patients with mild PsA and non-converters;
3. PsA disease activity—to identify markers associated with 3 levels of PsA disease activity.

### The structure of the PsC group

#### Baseline converters vs. baseline non-converters

**Fig. 3:**
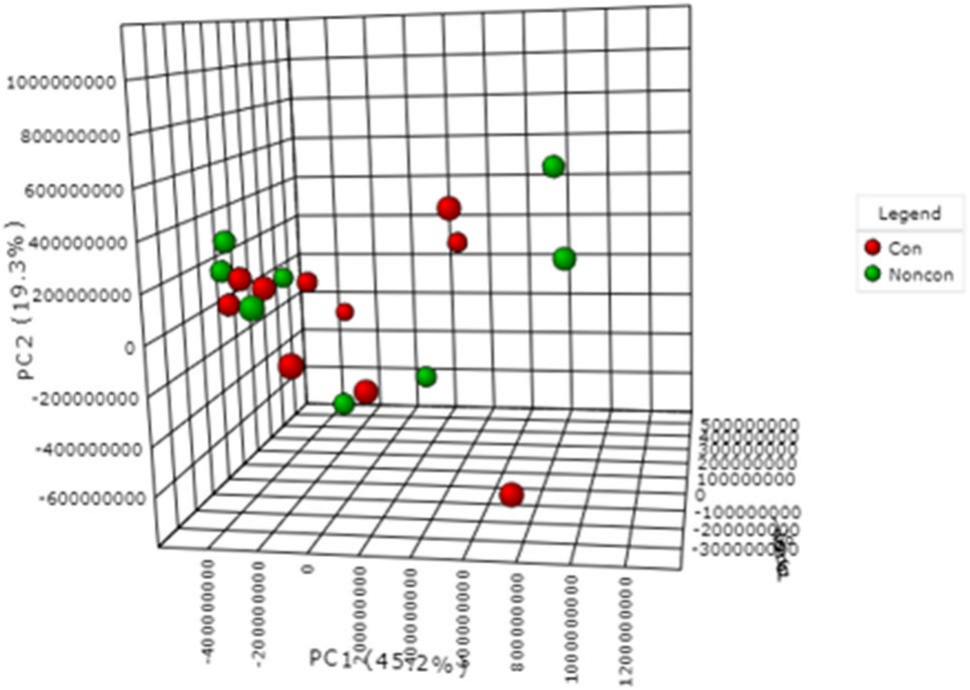
PCA (PC1: 45.2%, PC2: 19.3%, PC3: 9.6%) plot of patients with psoriasis who developed psoriatic arthritis prior to disease progression (baseline converters) and patients with psoriasis who had not developed psoriatic arthritis (baseline non-converters). Baseline converters and baseline non-converters are represented by red and green, respectively. Data were obtained via negative mode acquisition and were similar to the results obtained in positive mode acquisition (data not shown)

No statistically significant differences were found between baseline converters and baseline non-converters, which are represented in green and red, respectively, in Fig. 3. Detailed information for these patients related to the duration (in years) of psoriasis, PASI score, comorbidities, age, sex, and medication can be found in Online Resource 1—Table 2.

#### Baseline converters vs. follow-up converters

**Fig. 4:**
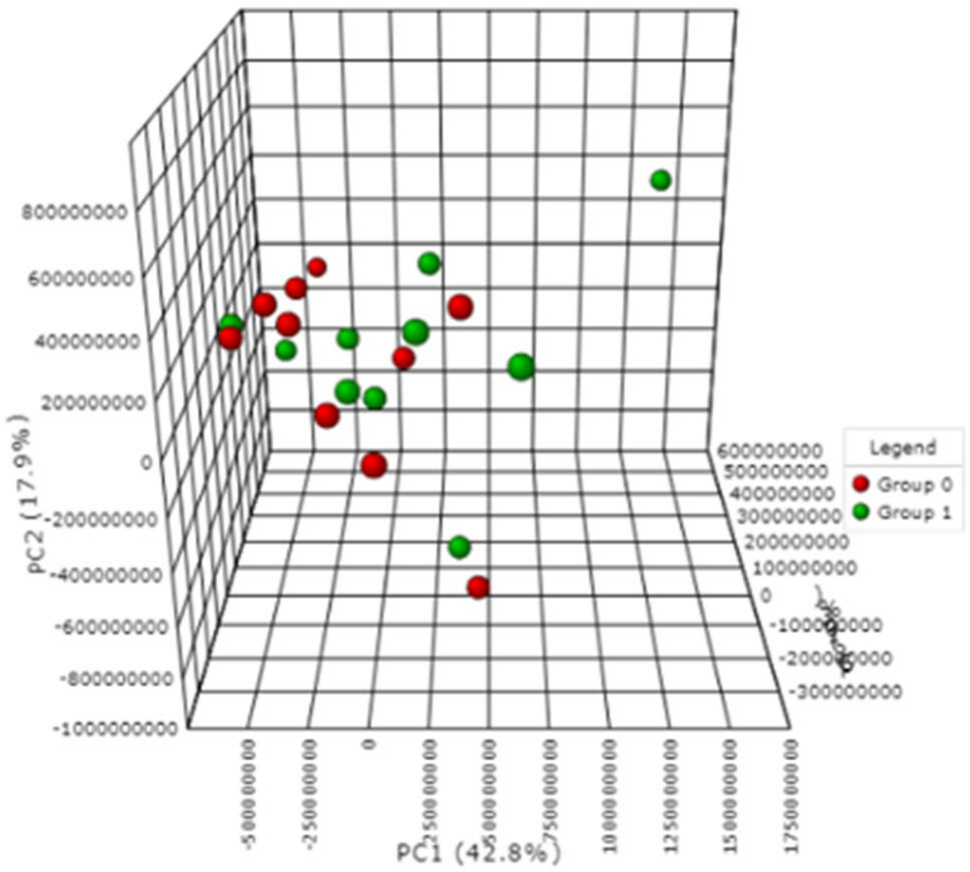
PCA (PC1: 42.8, PC2: 17.9%, PC3: 10.3%) plot of a paired analysis of converter (PsC) patients who had psoriasis without psoriatic arthritis at baseline (baseline converters) and developed psoriatic arthritis (follow-up converters). Baseline converters and follow-up converters are represented on the plot by red and green, respectively. Data were obtained via negative mode acquisition and were similar to the results obtained in positive mode acquisition (data not shown)

Subsequent comparisons were made to examine the metabolic changes occurring during the transition from PsC (baseline converter) to the development of PsA (follow-up converter). However, as the PCA plot in Fig. 4 illustrates, a paired analysis (Wilcoxon ranks test) revealed no statistically significant differences between baseline converters (shown in red) and follow-up converters (shown in green). Detailed patient information related to the duration (in years) of psoriasis, duration (in years) of PsA, PASI score, comorbidities, age, sex, and psoriasis-related medication can be found in Online Resource 1—Table 3.

### The relationship between PsC and PsA

#### Healthy controls vs. PsC vs mild PsA

Online Resource 1—Fig. 2 and Online Resource 1—Fig. 3 show that there exists no discrimination between patients with psoriasis who develop PsA (follow-up converters), patients with psoriasis who do not develop PsA (follow-up non-converters) and healthy controls (Online Resource 1—Fig. 3). Additionally, no differences were observed between follow-up converters, mild PsA, and healthy controls (Online Resource 1—Fig. 2).

#### PsC vs PsA

**Fig. 5:**
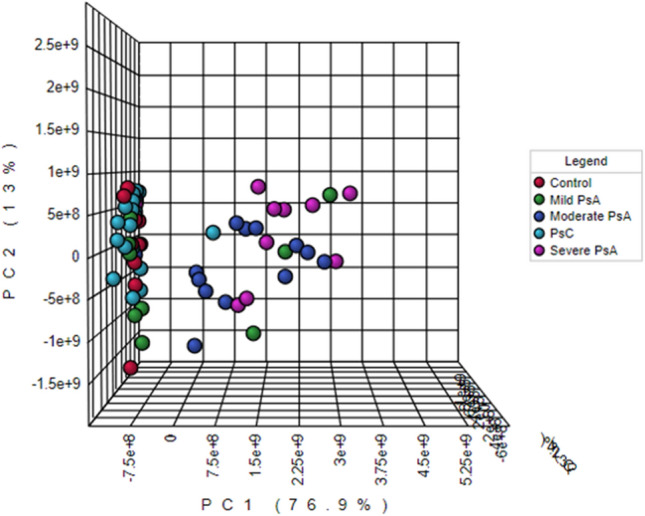
PCA (PC1: 76.9, PC2: 13%, PC3: 2.4%) plot of healthy volunteers (control; red) and patients with psoriasis prior to conversion or non-conversion, at baseline (PsC; turquoise). The plot also distinguishes between PsA patients based on level of disease activity (mild PsA-green; moderate PsA-dark blue; severe PsA-magenta). Data were obtained via negative mode acquisition and were similar to the results obtained in positive mode (data not shown)

It is abundantly clear from Figs. 2 and 5—which explicitly highlights PsA disease activity in comparison to healthy controls and patients with PsC—that the PsA group dominates the PCA plot. Specifically, compared to the healthy controls and the PsC group, the moderate and severe PsA groups contain the largest distribution of samples across the plot, accounting for 76.9% of the variation seen in the first principal component (PC1) (Fig. 5). Given the considerable variation observed among the PsA group, further investigation was conducted on the PsA group structure.

### PsA group disease activity

**Fig. 6:**
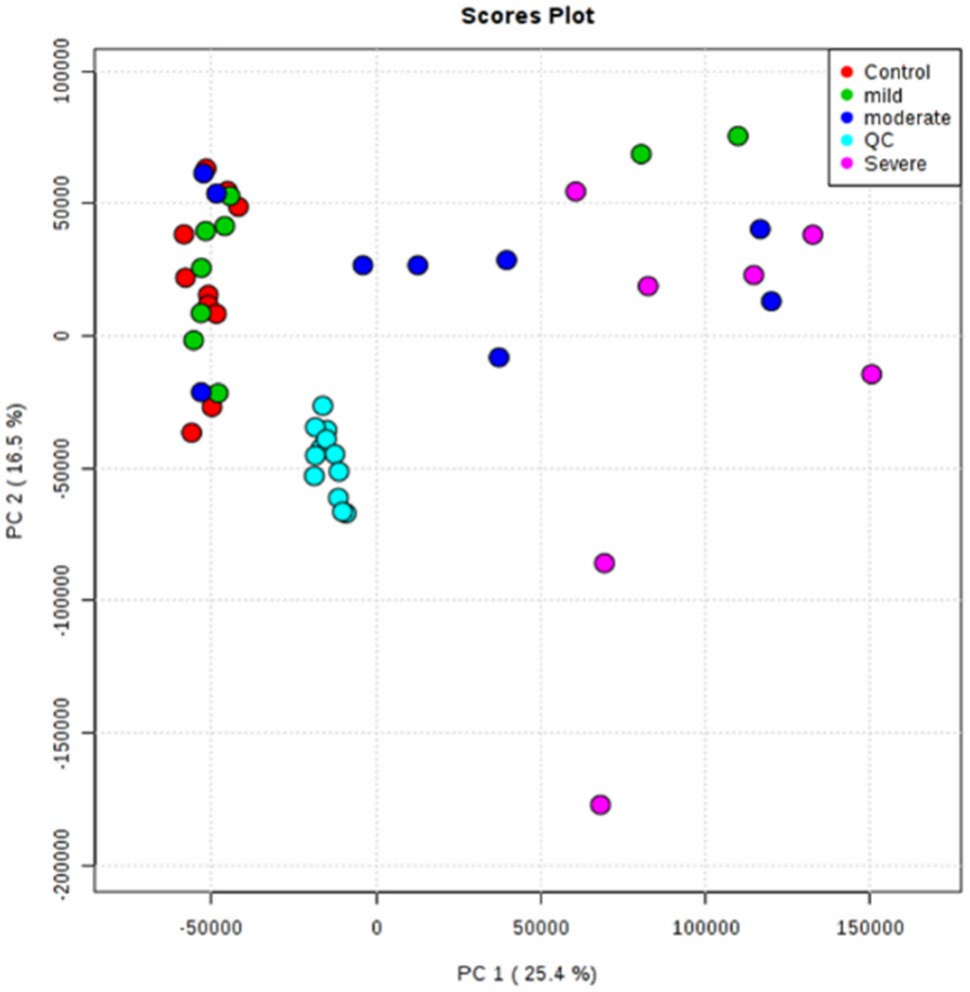
PCA (PC1:25.4%, PC2:16.5%) plot of healthy individuals (Ctrls) and patients with varying degrees of psoriatic arthritis (PsA) activity. The control group is represented by red, while patients with mild, moderate, and severe PsA are represented by green, dark blue, and magenta, respectively. Data were obtained via positive mode acquisition. Data obtained via negative mode acquisition can be found in Online resource 1—Fig. 7

The PCA plot in Fig. 6 shows a clear separation between PsA subgroups, such that as those with mild PsA tend towards the left side of the plot, those with severe PsA tend towards the right side of the plot, and those with moderate PsA are distributed among and between these two groups. Detailed information for these PsA patients related to the duration (in years) of psoriasis, duration (in years) of PsA, comorbidities, age, sex, and other non-psoriasis-related medication can be found in Online Resource 1—Table 4.

### Statistically significant features across patient groups

Statistical analysis via univariate analysis yielded 10 statistically significant features of note across the three PsA groups (mild, moderate and severe), which were putatively identified using xMSAnnotator for negative mode data while XCMS online was used to tentatively identify seven statistically significant features differentiating healthy individuals from patients with severe PsA for the positive mode data. Both METLIN and HMDB were used in conjunction with xMSAnnotator and XCMS online. See Online Resource 3—Table 1 and Online Resource 3—Table 2 for a complete list of annotated compounds along with their associated annotation confidence levels, statistical parameters and LC-HRMS parameters for both positive and negative modes, respectively.

Multivariate analysis of baseline converters and severe PsA patients via O-PLS-DA yielded a model with the acceptable criteria of 0.94 (R^2^) and 0.78 (Q^2^) in positive mode and 0.94 (R^2^) and 0.87(Q^2^) in negative mode (data for validation is provided in Online Resource 1—Fig. 4). The features with a variable importance in projection (VIP) score of > 1 that had the most significant influence on the model were putatively identified as 3-hydroxytetradecanedioic acid and 3-hydroxydodecanedioic acid for positive and negative mode, respectively. A list of the other features with VIP scores of > 1, their respective parameters, and their possible biochemical importance is provided in Online Resource 3—Tables 3 and 4. In the case of positive mode, two compounds (3-hydroxytetradecanedioic acid and L-phenylalanine) showed statistical significance between some of the considered data sets and are discussed in more detail in Online Resource 3—S1. For negative mode, four compounds (3-hydroxydodecanedioic acid, androsterone sulfate, 6-hydroxypentadecanedioic acid and 2-hydroxydecanedioic acid (or 3-hydroxysebacic acid, or cis-4-hydroxycyclohexylacetic acid) showed statistical significance between some of the considered data sets as discussed in more detail in Online Resource 3—S1. Ultimately, all putatively identified compounds were found to increase in relation to disease activity, with mild PsA patients exhibiting the lowest levels, moderate PsA patients exhibiting much higher levels, and severe PsA patients exhibiting the highest levels of the three groups. An example of this trend is demonstrated in Online Resource 1—Fig. 5 specifically for dodecanedioic acid.

### Putatively identified metabolite classes from PsC patients, PsA patients, and healthy controls

Several classes of endogenous compounds were extracted and detected with the use of TFME-LC-HRMS method, and then tentatively identified with the use of xMSAnnotator and HMDB (Online Resource 3—Tables 5 and 6). The structural annotation of molecular features (metabolites) detected in positive and negative modes revealed the changes in the composition of endogenous compounds among the analyzed groups of patients and healthy controls. Putative identification of the presented features enabled selection of compounds with important biological functions, such as different lipids species, steroid hormones, and amino acids among others. In addition, a number of different eicosanoids, including prostaglandins, leukotrienes and also derivatives of eicosapentaenoic acid, docosahexaenoic acid were detected in serum samples of patients with moderate and severe PsA activity, but not with mild PsA. The major observation, confirming the results obtained from multivariate analysis, was that of significantly higher levels of fatty acids, namely 3-hydroxytetradecanedioic acid and 3-hydroxydodecanedioic acid in three groups of PsA patients (Online Resource 3—Table 2). Serum levels of dopamine quinone, a product of dopamine oxidation (Online resource 3—Table 5), was significantly elevated in patients with PsA, but not in converters and non-converters neither at baseline nor follow-up. Analysis of peak areas of other detected metabolites did not reveal any significant differences in the level of those compounds in the analyzed groups of patients and healthy controls.

## Discussion

### Study objective 1: the conversion from psoriasis to PsA

#### Baseline converters vs. baseline non-converters

In this study, metabolic differences between patients classified as converters and non-converters at baseline as a means of possibly determining early markers of PsA disease development have been investigated. However, no significant differences were found to differentiate the two groups of patients. Given that both sets of patients have psoriasis and neither group was receiving treatments, this could suggest a very subtle onset of PsA (*this is discussed more in the following *Sect. “Baseline converters vs. follow-up converters”), further highlighting the challenges of early detection of PsA development using serum metabolomics.

#### Baseline converters vs. follow-up converters

Since at baseline, the converter group, according to the results presented herein, are not significantly different from baseline non-converters, strictly monitoring the differences between patients with psoriasis that eventually develop PsA may be more insightful. It is important to note however, that the follow-up samples were collected while patients were receiving treatment. These treatments may have corrected underlying inflammatory/metabolic changes and compounded by the small sample size, made it difficult to identify differences between the ‘before’ and ‘after’ profiles of psoriasis patients who develop PsA.

Another possible reason for the lack of differentiation between the baseline converters and their follow-up samples may be that the follow-up converters had only developed PsA relatively recently (less than 1 year; Online Resource 1—Table 3). As such, it is possible that allowing a longer time period before sampling post conversion may enable the identification of metabolites indicative of disease conversion. However, according to Fig. 2, Online Resource 1—Fig. 2, and the patient information for converters provided in Online Resource 1—Table 3, the follow-up converters (green), who are different from patients with mild PsA (blue), do not exhibit separation from patients with specifically mild PsA. Moreover, disease duration among mild PsA patients ranged from less than a year to 16 years, arguing against delayed sampling to identify markers of conversion. A closer examination between the PsC and PsA group is available in the following Sect. “Study objective 2: PsA activity in PsC patients”.

The lack of discrimination between the samples collected before and after conversion and between the baseline converters and non-converters may also indicate a small effect size (very subtle changes or differences) for both between-group comparisons. As such, it may be necessary to analyze a very large number of samples from each group before distinctive differences can be observed. Indeed, a post hoc power analysis showed (Online Resource 1—Fig. 6) that over 1000 serum samples would be required before any statistically significant differences (power ≥ 0.8) would be observable in the ‘before’ and ‘after’ conversion samples, as well as in the baseline samples for the converters and non-converters. It is therefore possible that the metabolic changes in the peripheral blood related to conversion are perhaps too subtle, which would potentially make serum an ineffective biological fluid for identifying metabolites predicting PsA development.

### Study objective 2: PSA activity in PsC patients

#### Healthy controls vs. PsC vs. mild PsA

No discrimination was found among patients with psoriasis (baseline converters and follow-up non-converters of the PsC group), patients with mild PsA, and healthy controls; an observation that has not been previously reported. This result was interesting given the number of metabolomic studies demonstrating visual differences between and among patients with psoriasis vs. patients with psoriatic arthritis vs. healthy individuals. Since the PsC group collectively appear to exhibit similar metabolic profiles to mild PsA with the lack of discrimination between these two groups, it suggests that patients within these two groups may in fact be similar. This observation emphasizes the challenges in clearly identifying markers of PsA development. More importantly, it highlights the challenges in accurately identifying early markers of conversion as this information can be easily obscured by the grouping of all PsA patients collectively, a limitation presented by past studies. The majority of baseline converters and mild PsA patients had a PASI score of ≤ 10, indicating milder psoriasis. As such, it follows that no discrimination may be discerned amongst these three groups. This is consistent with the results of Li et al.’s (Li et al., 2017) analysis of serum samples from psoriasis vulgaris patients with varying degrees of psoriasis severity wherein patients with mild psoriasis were similar to the controls and significantly different from patients with severe psoriasis. Ultimately, the indiscernible zone between PsC patients and mild PsA patients presents a clear need for a closer investigation of metabolic markers of conversion with a much larger and more defined sample cohort.

#### PsC vs PsA

Since it is abundantly clear from Figs. 2 and 5 that the PsA group dominates the PCA plot, this result may suggest that the detecting metabolomic abnormalities related to conversion from PsC to PsA is driven by PsA disease activity.

### Study objective 3: PsA group disease activity

#### Confounding factors

Considering the patient information described in Online Resource 1—Table 4, and based on the results shown in Fig. 6, it is clear that neither the duration of psoriasis prior to developing PsA nor the duration of PsA is a predictor of disease conversion or disease activity, respectively. While psoriasis is related to a number of associated comorbidities, such as cardiovascular disease, obesity, insulin resistance, and metabolic syndrome, and the observed patients may be at a risk of other age-related comorbidities (≥ 40 years old), the differentiating pattern on the plot appears to be independent of these factors rather than confounded by them, since all three groups contain patients with and without a range of comorbidities. Additionally, disease severity appears to be independent of sex or age. However, the small sample size makes it difficult to make any firm conclusions and will be the subject of future studies.

#### Cluster deviants

It is worth noting that one or two patients from each group were removed from statistical analysis due to being outliers. Conversely, some patients fell outside the majority of their cluster but were not removed because they did not statistically classify as outliers. For example, as demonstrated in Fig. 6, two patients with mild PsA (green) and two patients with moderate PsA (dark blue) are located on the extreme right side of the plot with the severe PsA patients (pink), while three moderate PsA patients (dark blue) are located on the left side of the plot with the majority of the mild PsA patients (green) and healthy controls (red). These inconsistent patterns may suggest that the current clinical methods being used to evaluate PsA disease activity, which are based on clinical assessment of joint inflammation (tenderness, swelling) may not be accurate. This highlights the challenges of disease categorization, even with very stringent phenotyping protocols and emphasizes the need to use biomarkers, such as metabolic markers or ultrasonography as a complementary source of information for assessing disease activity.

### Statistically significant features across patient groups

Based on the performed TFME-LC-HRMS analysis along metabolite annotation with the use of xMSAnnotator, HMDB and METIN database, we putatively identified 1,11-undecanedicarboxylic acid, an unusual fatty acid previously found in individuals suffering from peroxisomal disorders wherein the enzymes within the peroxisome that are responsible for the breakdown/digestion of long chain fatty acids by the process of oxidation, malfunction (Korman et al., 2000). This also corroborates previous metabolomics investigations that focussed solely on the differences between patients with psoriasis and healthy individuals (Ottas et al., 2017). Other tentatively identified compounds such as S-aminomethyldihydrolipoamide which was annotated in both the positive and negative mode data in this study, is associated with the serine and threonine metabolism pathways. This correlated well with research presented by Kamleh et al*.* (Kamleh et al., 2015) in which these pathways were discovered to be affected in psoriatic patients, resulting in higher serine and threonine levels when compared to healthy individuals. Elevated levels of 3-hydroxytetradecanedioic acid, characteristically higher in patients with severe PsA in comparison to baseline converters, have been implicated in the inhibition of CoA, which is an enzyme involved in fatty acid biosynthesis. Moreover, 3-hydroxydodecanedioic acid, also elevated in severe PsA patients, has also been linked to inhibited fatty acid oxidation (Bergoffen et al., 1993; Matsumoto et al., 1991; Okajima et al., 2002). Interestingly, the majority of the other VIPs, especially those identified in negative mode, were tentatively identified as various dicarboxylic and carboxylic acid derivatives [i.e. 3-hydroxysebacic acid implicated as a result of fatty acid beta-oxidation defects (Muth et al., 2003)], thus indicating a potentially strong correlation between the dysregulation of the fatty acid or lipidome profile and disease activity.

However, further investigation is required to confirm these findings due to the challenges associated with correlating circulating endogenous compounds, especially organic acids, with specific biochemical processes. For example, elevated levels on their own may represent a non-specific response, while ratios of various kinds of metabolites like these organic acids may indeed be more informative (Bergoffen et al., 1993).

### Metabolite fingerprinting of serum samples from PsC patients, PsA patients, and healthy controls

Untargeted metabolomics of serum samples was used to provide insight into the profile of low molecular weight molecules as potential biomarkers of altered tissue metabolism during psoriasis disease conversion. In the current study, the metabolomes of patients with PsA exhibited disturbances in lipid metabolism, resulting in increased levels of 3-hydroxytetradecanedioic acid and 3-hydroxydodecanedioic acid, probably in response to peroxidation processes. The results of the presented metabolomics studies strongly suggest that imbalance in lipid profiles might contribute to PsA disease activity. The relationship between lipid metabolism and arthritis have been previously reported, and alterations in the level of several endogenous components (e.g. amino acids, lipids) identified as signalling molecules and cellular messengers were observed in previous metabolomic studies (Armstrong et al., 2014; Coras et al., 2019; Guma et al., 2016; Jiang et al., 2015). Armstrong et al. (Armstrong et al., 2014) used a global metabolomics approach to compare circulating metabolites in serum samples from patients with psoriasis or PsA and healthy controls. The study reported that patients with PsA had an increased level of lignoceric acid and a decreased level of α-ketoglutarate compared to patients with psoriasis. Armstrong et al. (2014) also observed decreased level of glutamine (Gln) in patients with psoriasis (but not PsA) in comparison to healthy controls. In our study however, Gln was detected solely in a group of patients with severe PsA. The presence of Gln in the serum samples of severe PsA patients may suggest protein degradation within cells. It may also suggest the impact of on-going inflammation or the effects of administered drugs on the occurrence of the free from of this amino acid (Online Resource 3—Table 6). Contrary, the application of the TFME device facilitated the extraction of a unique metabolite—dopamine quinone—not previously reported in psoriasis-related studies (Online Resource 3—Table 5). It was observed that oxidation or auto-oxidation of dopamine caused the formation of quinone metabolites that may evoke neurotoxicity and/or apoptotic or non-apoptotic cell death (Miyazaki & Asanuma, 2009; Monzani et al., 2019). The detected level of a feature putatively identified as dopamine quinone, similarly, to selected fatty acids, was also significantly elevated in PsA patients. As previously reported in several studies, TF-SPME is an important tool in the analysis of lipidome in physiological and pathological conditions as it facilitates extraction and monitoring of different lipids (Birjandi et al., 2017; Reyes-Garcés & Gionfriddo, 2019). Elevated levels of many lipid species, mainly medium- and long-chain fatty acids were dysregulated in patients with different levels of PsA disease activity. The dysregulation may be related to pathological conditions, including stress or inflammation, whereas the increased number of different triglycerides in patients with severe PsA may indicate potential imbalance in energy metabolism. In addition, the elevated level of multiple eicosanoids with either pro- or anti-inflammatory properties, which is in line with the previous studies (Coras et al., 2019), may also be related with PsA disease activity. Although the role of those eicosanoids in the pathogenesis of joint inflammation in PsA has not been confirmed, performed analysis of the metabolome of PsA patients indicated that the significant differences in the number and composition of eicosanoids occur mainly in patients with moderate and severe PsA. However, the observed differences at the metabolite level may not only be related to PsA disease activity alone, but also could result from comorbidities and pharmacotherapy. Therefore, further and more comprehensive analysis involving larger sample size from prospective cohorts of patients are needed.

## Conclusions and future directions

In this study, an attempt was made to determine biomarkers for the following: (1) the conversion from psoriasis to PsA; (2) differentiating PsA from PsC; and (3) PsA disease activity. Rapid sampling with the use of TF-SPME technique along with LC-HRMS analysis facilitated the assessment of PsA disease activity and pathophysiology by way of untargeted metabolomics fingerprinting of serum samples obtained from a wide range of psoriasis-related patients and healthy controls. The heterogeneity of the patient data presented herein (i.e., the large ranges in age, duration of psoriasis, duration of PsA, sex imbalances in some cases, range of treatments, and comorbidities) and the relatively small sample sizes (n = 10 for each group) made it difficult to exclude samples for the purpose of producing more uniform data sets, which are customary for statistical comparisons of patient groups in clinical applications. Nevertheless, the preliminary findings were interesting, as they showed no apparent trends or influence of potential confounding factors (comorbidities and treatment) of the patients’ biochemical parameters on the patterns observed via multivariate analysis. Despite this, the observed differences at the metabolite level may be related not only to PsA disease activity, but also could result from (1) comorbidities; or (2) can be a result of pharmacotherapy. Therefore, further and more comprehensive analysis involving larger sample cohorts of patients are needed. In contrast to previously reported studies, this is the first study that provides a closer examination of circulating metabolites associated with PsA activity by separately analyzing the metabolomic profile of PsA patients with differing levels of arthritis activity (mild, moderate, and severe). The observed differences at the metabolite level, while could be due to an array of other factors as stated above, are seemingly related to PsA disease activity specifically since a pattern emerges differentiating mild, moderate and severe even though all patients within the PsA group are suffering some type of comorbidity and/or are enduring a combination of pharmacotherapies. Moreover, the main alterations in the number of metabolites are observed in moderate and severe groups. Based on the data presented herein, it is apparent that dysregulated fatty acid metabolism comprising synthesis or degradation of various fatty acids, is strongly associated with PsA disease activity as many of these features differentiated baseline converters (PsC) from severe PsA with elevated levels in the latter. This pattern is also more evident for PsA pathology given the increasing levels of important fatty-acid-related features across the PsA group with the lowest levels in mild PsA and the highest levels in severe PsA. Furthermore, there are at least 20 statistically significant features associated with PsA activity, while there are no statistically significant features that differentiate between patients with psoriasis prior to and post conversion to PsA. This particular finding could suggest that, while serum may be a useful matrix for finding markers of PsA severity/activity, the differences in terms of PsA conversion from psoriasis may be too subtle to generate a significant and observable effect. Besides, in this data set, follow-up converter samples were taken from patients who started receiving treatment which could normalize metabolic abnormalities characteristic of PsA disease thereby obscuring the real important differences between baseline and follow-up conversion. Other limitations of the study worth mentioning presented by SPME, is the fact that while the use of more agreeable mixtures of sorbent particles for the extraction phase improves the coverage of hydrophilic compounds, their extraction currently remains poor—albeit a limitation experienced by many other sample preparation approaches as well. For SPME however, this can be overcome by the development of more polar extraction phases that are suitable for aqueous matrices like serum. Moreover, this preliminary study was also limited by the use of a single reversed-phase chromatographic method, restricting assessment of a broad range of other classes of compounds which could otherwise prove very useful.

As a next stage of biomarkers studies, we propose the use of synovial fluid for further metabolic analyses. As the joint fluid—synovial fluid—is found closest to the origin of PsA disease manifestation, it can likely provide a better indication of discriminating metabolic biomarkers, specifically for disease conversion. Since collection of synovial fluid may be quite difficult, it would be beneficial to develop a protocol for in vivo SPME sampling, as this would provide a minimally invasive approach for obtaining potentially more useful biological material directly from the source, while avoiding removal/collection from the patient. Furthermore, an in vivo SPME technique would enable sampling and sample preparation to be combined into one step, drastically minimizing the number of analytical steps required to obtain the final extract, thus preserving the initial integrity of the true metabolome (Bojko et al., 2014; Reyes-Garcés & Gionfriddo, 2019; Reyes-Garcés et al., 2019). Finally, while this preliminary data provides a good direction for future research, due to the relatively small sample sizes used, we suggest that a larger cohort follow-up study be conducted to confirm the findings. This expanded study should not only increase the number of patients examined, but investigate patients not receiving treatment, utilize parallel in vivo skin-lesion analysis, in vivo synovial fluid analysis, include targeted fatty acid profiling and expand metabolome coverage via exploration of other chromatographic methods amenable to other classes of metabolites like performing lipidome fingerprinting and/or using HILIC for more polar metabolites. These additional steps would provide a more supplemented and comprehensive approach to the metabolomics of PsD.

## Supplementary Information

Below is the link to the electronic supplementary material.

Supplementary file1 (PPTX 576 kb)

Supplementary file2 (DOCX 29 kb)

Supplementary file3 (DOCX 79 kb)

## Data Availability

All data generated or analyzed during this study are included in this published article and its supplementary information files.
