# Supplementary material for: Serum metabolic fingerprinting of psoriasis and psoriatic arthritis patients using solid-phase microextraction—liquid chromatography—high-resolution mass spectrometry
Source: Metabolomics. 2021 Jun 16;17(7):59. doi: 10.1007/s11306-021-01805-3 (PMC8211611; doi:10.1007/s11306-021-01805-3)
Supplement: Supplementary file 1 — Supplementary file1 (PPTX 576 kb) [file 11306_2021_1805_MOESM1_ESM.pptx]

## Slide 1
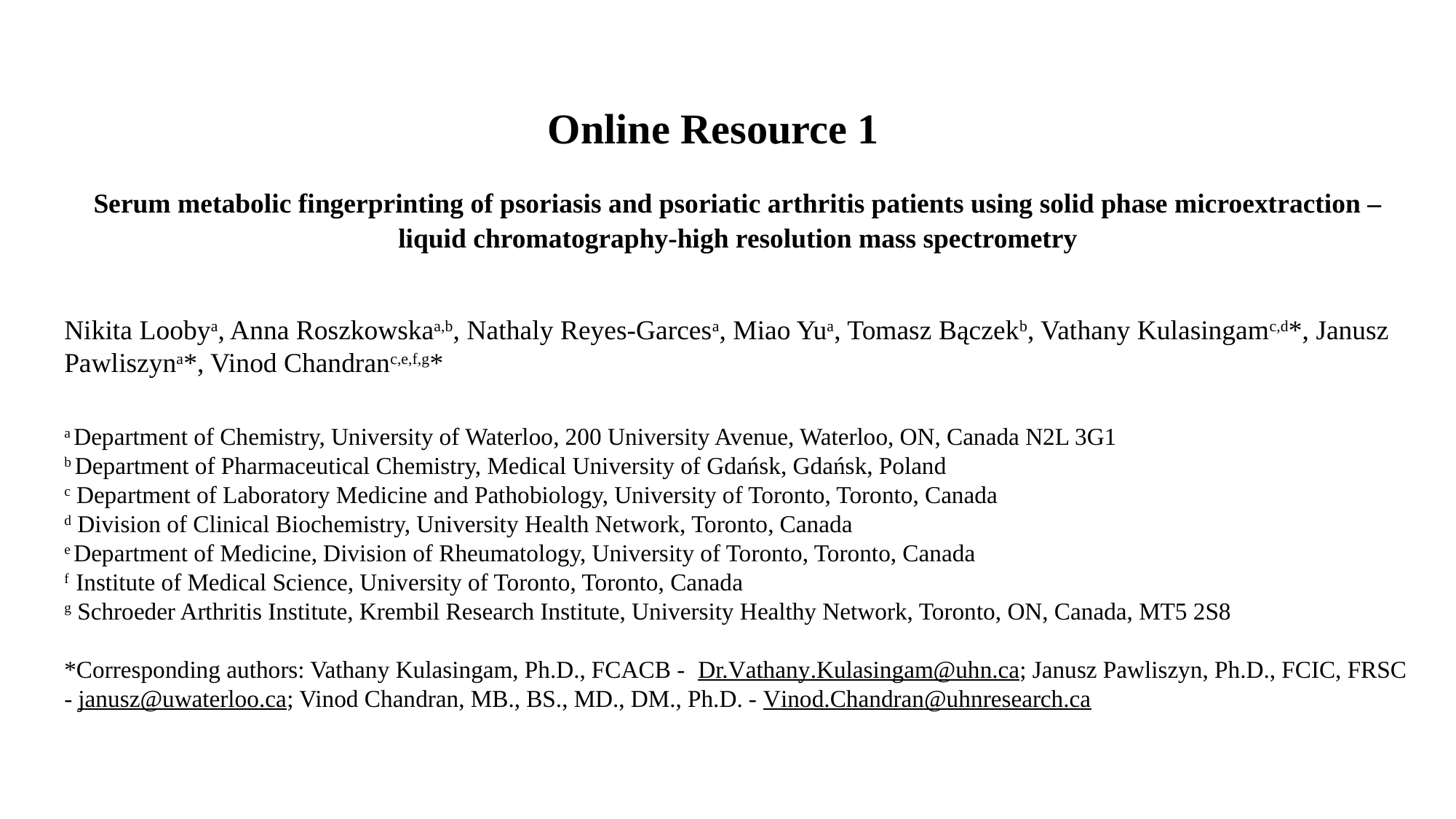

Online Resource 1
Serum metabolic fingerprinting of psoriasis and psoriatic arthritis patients using solid phase microextraction – liquid chromatography-high resolution mass spectrometry
Nikita Loobya, Anna Roszkowskaa,b, Nathaly Reyes-Garcesa, Miao Yua, Tomasz Bączekb, Vathany Kulasingamc,d*, Janusz Pawliszyna*, Vinod Chandranc,e,f,g*
a Department of Chemistry, University of Waterloo, 200 University Avenue, Waterloo, ON, Canada N2L 3G1
b Department of Pharmaceutical Chemistry, Medical University of Gdańsk, Gdańsk, Poland
c Department of Laboratory Medicine and Pathobiology, University of Toronto, Toronto, Canada
d Division of Clinical Biochemistry, University Health Network, Toronto, Canada
e Department of Medicine, Division of Rheumatology, University of Toronto, Toronto, Canada
f Institute of Medical Science, University of Toronto, Toronto, Canada
g Schroeder Arthritis Institute, Krembil Research Institute, University Healthy Network, Toronto, ON, Canada, MT5 2S8
*Corresponding authors: Vathany Kulasingam, Ph.D., FCACB - Dr.Vathany.Kulasingam@uhn.ca; Janusz Pawliszyn, Ph.D., FCIC, FRSC - janusz@uwaterloo.ca; Vinod Chandran, MB., BS., MD., DM., Ph.D. - Vinod.Chandran@uhnresearch.ca

## Slide 2
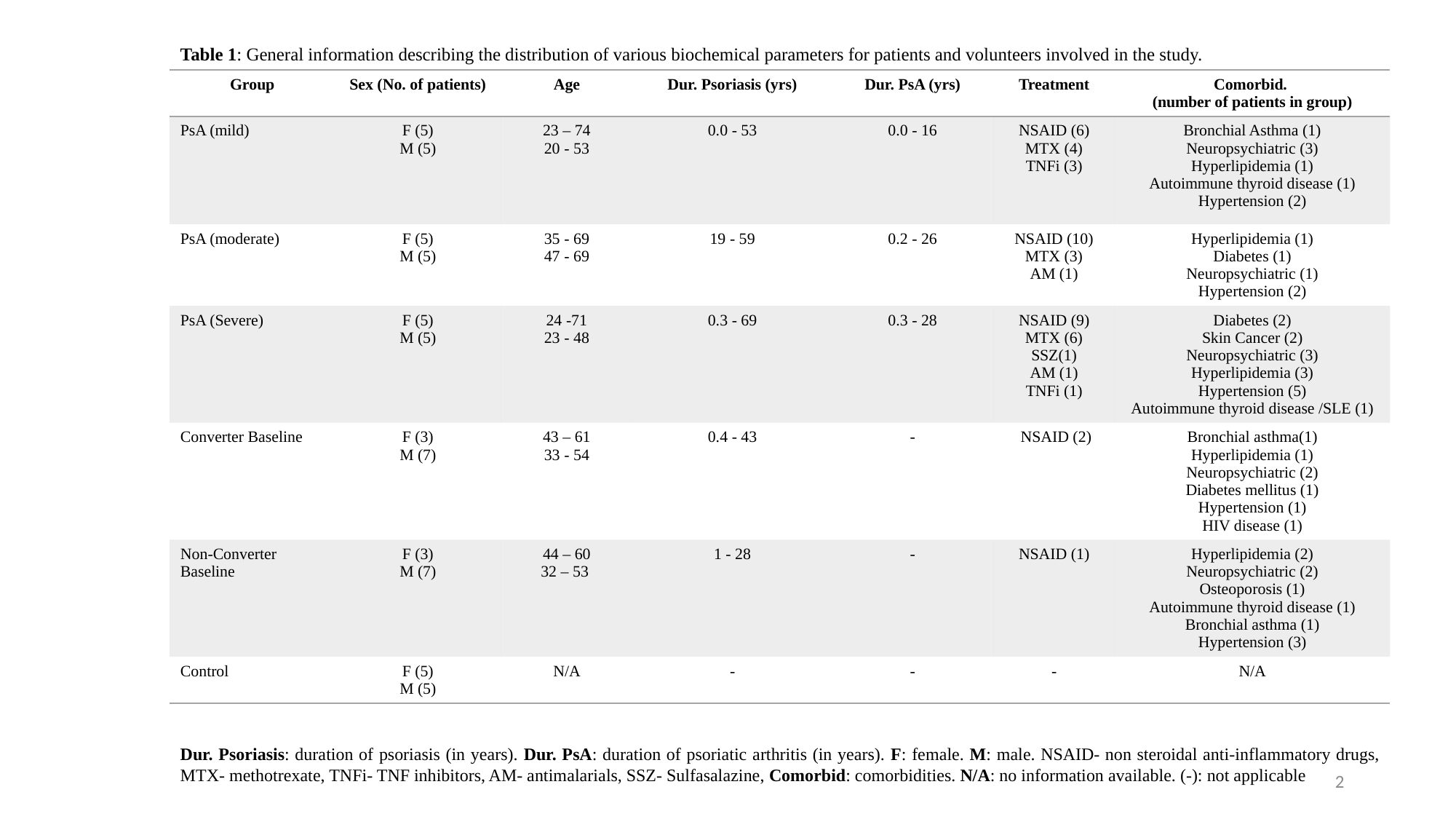

Table 1: General information describing the distribution of various biochemical parameters for patients and volunteers involved in the study.
| Group | Sex (No. of patients) | Age | Dur. Psoriasis (yrs) | Dur. PsA (yrs) | Treatment | Comorbid. (number of patients in group) |
| --- | --- | --- | --- | --- | --- | --- |
| PsA (mild) | F (5) M (5) | 23 – 74 20 - 53 | 0.0 - 53 | 0.0 - 16 | NSAID (6) MTX (4) TNFi (3) | Bronchial Asthma (1) Neuropsychiatric (3) Hyperlipidemia (1) Autoimmune thyroid disease (1) Hypertension (2) |
| PsA (moderate) | F (5) M (5) | 35 - 69 47 - 69 | 19 - 59 | 0.2 - 26 | NSAID (10) MTX (3) AM (1) | Hyperlipidemia (1) Diabetes (1) Neuropsychiatric (1) Hypertension (2) |
| PsA (Severe) | F (5) M (5) | 24 -71 23 - 48 | 0.3 - 69 | 0.3 - 28 | NSAID (9) MTX (6) SSZ(1) AM (1) TNFi (1) | Diabetes (2) Skin Cancer (2) Neuropsychiatric (3) Hyperlipidemia (3) Hypertension (5) Autoimmune thyroid disease /SLE (1) |
| Converter Baseline | F (3) M (7) | 43 – 61 33 - 54 | 0.4 - 43 | - | NSAID (2) | Bronchial asthma(1) Hyperlipidemia (1) Neuropsychiatric (2) Diabetes mellitus (1) Hypertension (1) HIV disease (1) |
| Non-Converter Baseline | F (3) M (7) | 44 – 60 32 – 53 | 1 - 28 | - | NSAID (1) | Hyperlipidemia (2) Neuropsychiatric (2) Osteoporosis (1) Autoimmune thyroid disease (1) Bronchial asthma (1) Hypertension (3) |
| Control | F (5) M (5) | N/A | - | - | - | N/A |
Dur. Psoriasis: duration of psoriasis (in years). Dur. PsA: duration of psoriatic arthritis (in years). F: female. M: male. NSAID- non steroidal anti-inflammatory drugs, MTX- methotrexate, TNFi- TNF inhibitors, AM- antimalarials, SSZ- Sulfasalazine, Comorbid: comorbidities. N/A: no information available. (-): not applicable
2

## Slide 3
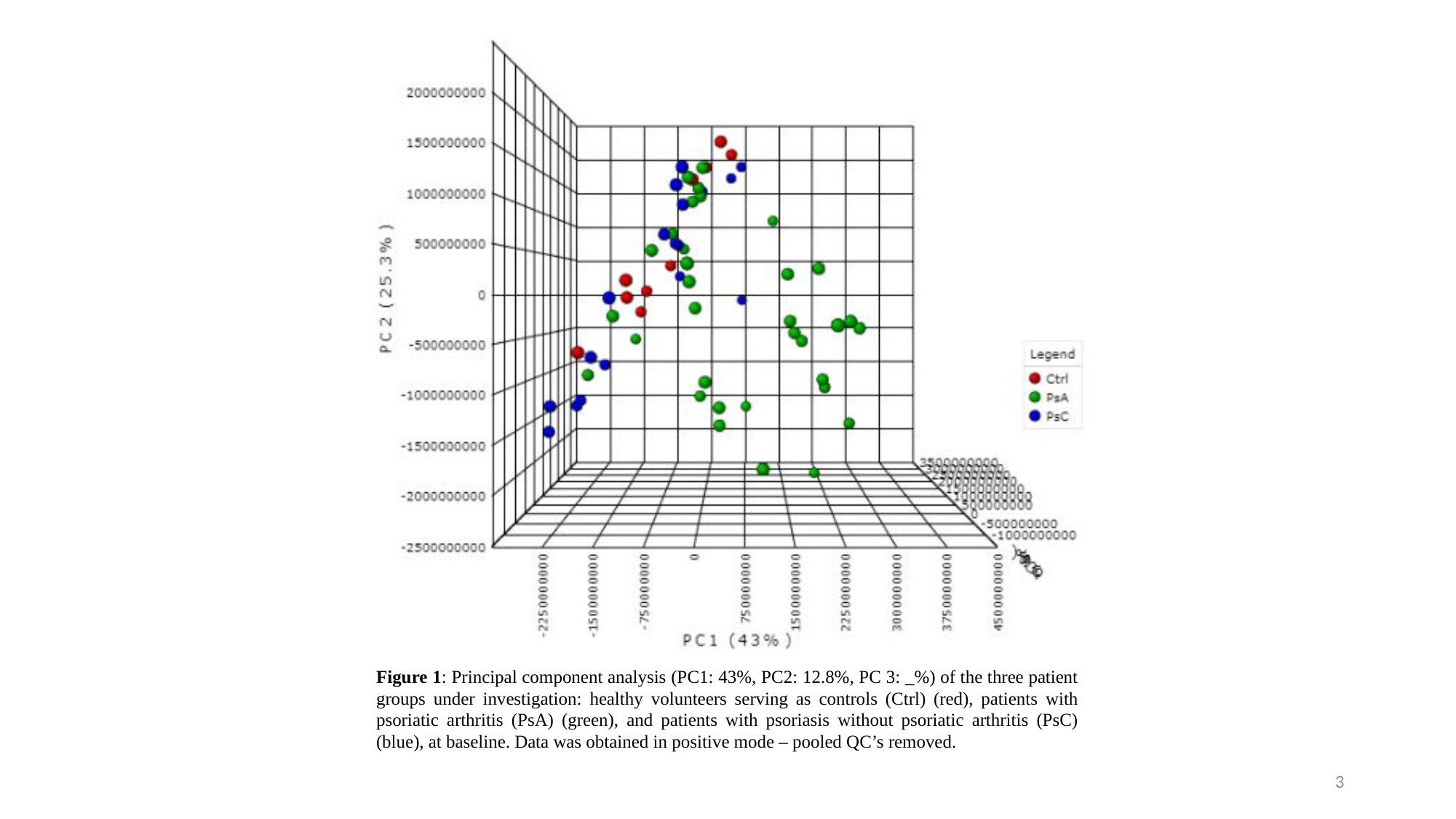

Figure 1: Principal component analysis (PC1: 43%, PC2: 12.8%, PC 3: _%) of the three patient groups under investigation: healthy volunteers serving as controls (Ctrl) (red), patients with psoriatic arthritis (PsA) (green), and patients with psoriasis without psoriatic arthritis (PsC) (blue), at baseline. Data was obtained in positive mode – pooled QC’s removed.
3

## Slide 4
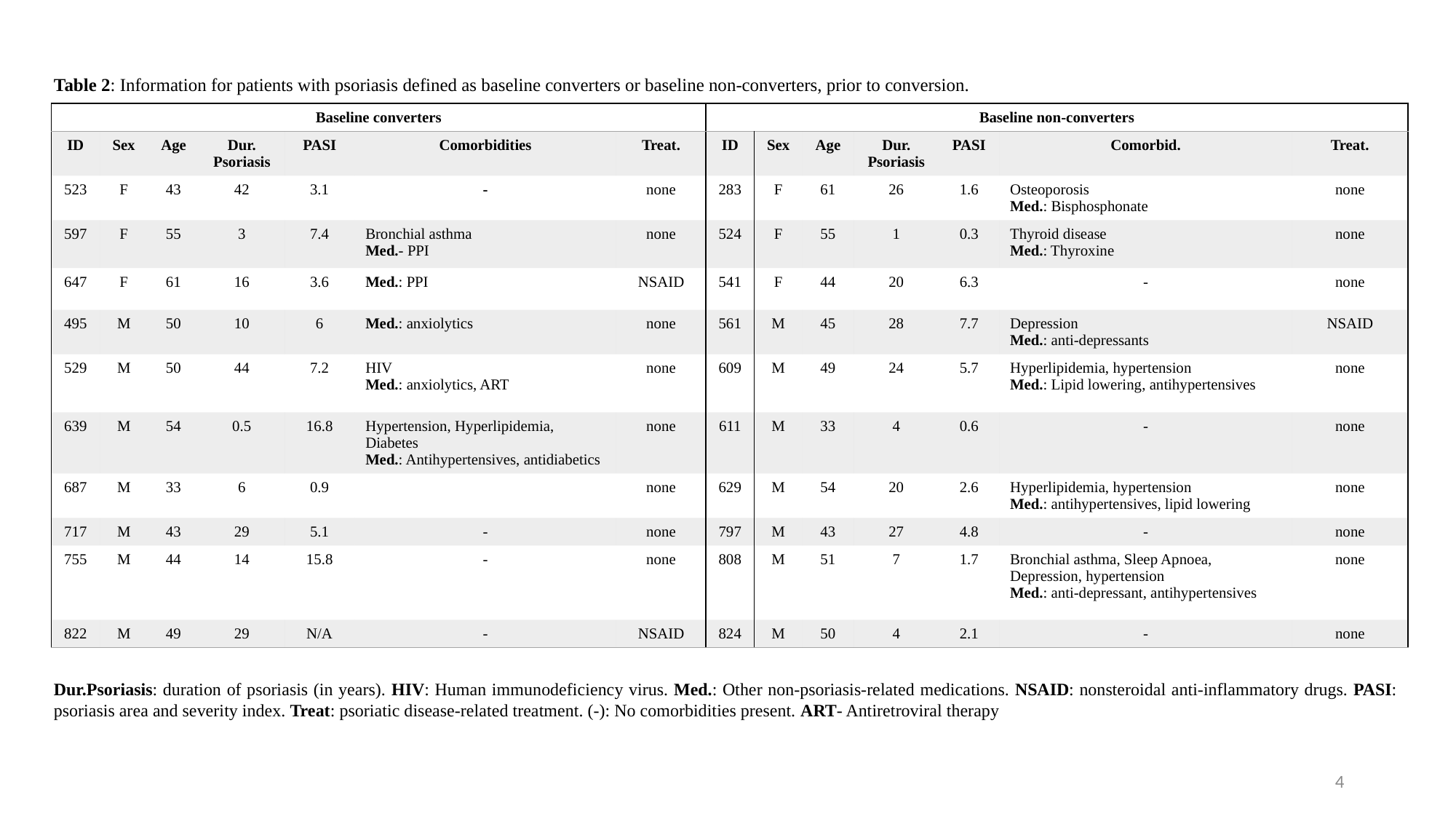

Table 2: Information for patients with psoriasis defined as baseline converters or baseline non-converters, prior to conversion.
| Baseline converters | | | | | | | Baseline non-converters | | | | | | |
| --- | --- | --- | --- | --- | --- | --- | --- | --- | --- | --- | --- | --- | --- |
| ID | Sex | Age | Dur. Psoriasis | PASI | Comorbidities | Treat. | ID | Sex | Age | Dur. Psoriasis | PASI | Comorbid. | Treat. |
| 523 | F | 43 | 42 | 3.1 | - | none | 283 | F | 61 | 26 | 1.6 | Osteoporosis Med.: Bisphosphonate | none |
| 597 | F | 55 | 3 | 7.4 | Bronchial asthma Med.- PPI | none | 524 | F | 55 | 1 | 0.3 | Thyroid disease Med.: Thyroxine | none |
| 647 | F | 61 | 16 | 3.6 | Med.: PPI | NSAID | 541 | F | 44 | 20 | 6.3 | - | none |
| 495 | M | 50 | 10 | 6 | Med.: anxiolytics | none | 561 | M | 45 | 28 | 7.7 | Depression Med.: anti-depressants | NSAID |
| 529 | M | 50 | 44 | 7.2 | HIV Med.: anxiolytics, ART | none | 609 | M | 49 | 24 | 5.7 | Hyperlipidemia, hypertension Med.: Lipid lowering, antihypertensives | none |
| 639 | M | 54 | 0.5 | 16.8 | Hypertension, Hyperlipidemia, Diabetes Med.: Antihypertensives, antidiabetics | none | 611 | M | 33 | 4 | 0.6 | - | none |
| 687 | M | 33 | 6 | 0.9 | | none | 629 | M | 54 | 20 | 2.6 | Hyperlipidemia, hypertension Med.: antihypertensives, lipid lowering | none |
| 717 | M | 43 | 29 | 5.1 | - | none | 797 | M | 43 | 27 | 4.8 | - | none |
| 755 | M | 44 | 14 | 15.8 | - | none | 808 | M | 51 | 7 | 1.7 | Bronchial asthma, Sleep Apnoea, Depression, hypertension Med.: anti-depressant, antihypertensives | none |
| 822 | M | 49 | 29 | N/A | - | NSAID | 824 | M | 50 | 4 | 2.1 | - | none |
Dur.Psoriasis: duration of psoriasis (in years). HIV: Human immunodeficiency virus. Med.: Other non-psoriasis-related medications. NSAID: nonsteroidal anti-inflammatory drugs. PASI: psoriasis area and severity index. Treat: psoriatic disease-related treatment. (-): No comorbidities present. ART- Antiretroviral therapy
4

## Slide 5
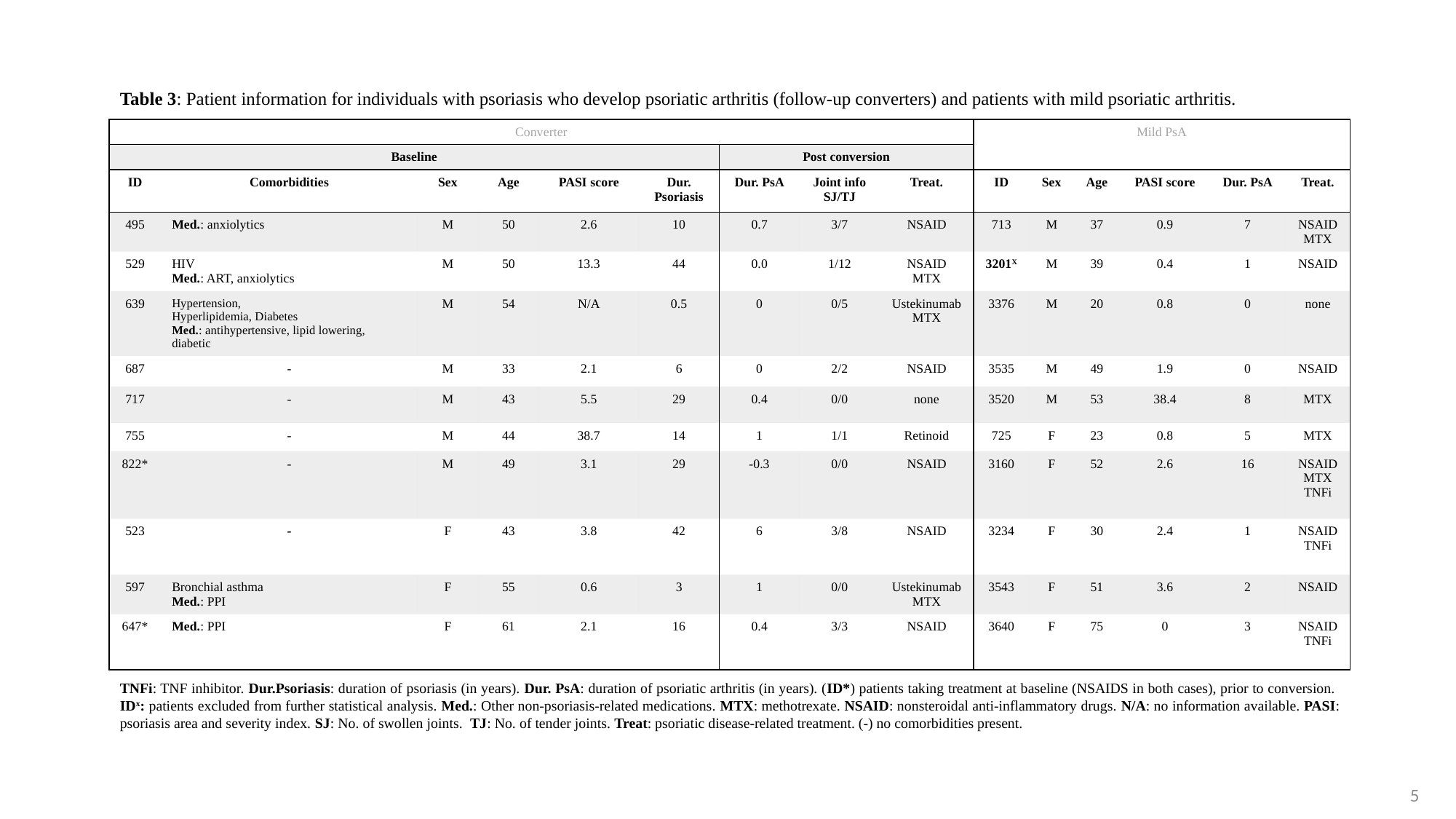

Table 3: Patient information for individuals with psoriasis who develop psoriatic arthritis (follow-up converters) and patients with mild psoriatic arthritis.
| Converter | | | | | | | | | Mild PsA | | | | | |
| --- | --- | --- | --- | --- | --- | --- | --- | --- | --- | --- | --- | --- | --- | --- |
| Baseline | | | | | | Post conversion | | | | | | | | |
| ID | Comorbidities | Sex | Age | PASI score | Dur. Psoriasis | Dur. PsA | Joint info SJ/TJ | Treat. | ID | Sex | Age | PASI score | Dur. PsA | Treat. |
| 495 | Med.: anxiolytics | M | 50 | 2.6 | 10 | 0.7 | 3/7 | NSAID | 713 | M | 37 | 0.9 | 7 | NSAID MTX |
| 529 | HIV Med.: ART, anxiolytics | M | 50 | 13.3 | 44 | 0.0 | 1/12 | NSAID MTX | 3201X | M | 39 | 0.4 | 1 | NSAID |
| 639 | Hypertension, Hyperlipidemia, Diabetes Med.: antihypertensive, lipid lowering, diabetic | M | 54 | N/A | 0.5 | 0 | 0/5 | Ustekinumab MTX | 3376 | M | 20 | 0.8 | 0 | none |
| 687 | - | M | 33 | 2.1 | 6 | 0 | 2/2 | NSAID | 3535 | M | 49 | 1.9 | 0 | NSAID |
| 717 | - | M | 43 | 5.5 | 29 | 0.4 | 0/0 | none | 3520 | M | 53 | 38.4 | 8 | MTX |
| 755 | - | M | 44 | 38.7 | 14 | 1 | 1/1 | Retinoid | 725 | F | 23 | 0.8 | 5 | MTX |
| 822\* | - | M | 49 | 3.1 | 29 | -0.3 | 0/0 | NSAID | 3160 | F | 52 | 2.6 | 16 | NSAID MTX TNFi |
| 523 | - | F | 43 | 3.8 | 42 | 6 | 3/8 | NSAID | 3234 | F | 30 | 2.4 | 1 | NSAID TNFi |
| 597 | Bronchial asthma Med.: PPI | F | 55 | 0.6 | 3 | 1 | 0/0 | Ustekinumab MTX | 3543 | F | 51 | 3.6 | 2 | NSAID |
| 647\* | Med.: PPI | F | 61 | 2.1 | 16 | 0.4 | 3/3 | NSAID | 3640 | F | 75 | 0 | 3 | NSAID TNFi |
TNFi: TNF inhibitor. Dur.Psoriasis: duration of psoriasis (in years). Dur. PsA: duration of psoriatic arthritis (in years). (ID*) patients taking treatment at baseline (NSAIDS in both cases), prior to conversion. IDx: patients excluded from further statistical analysis. Med.: Other non-psoriasis-related medications. MTX: methotrexate. NSAID: nonsteroidal anti-inflammatory drugs. N/A: no information available. PASI: psoriasis area and severity index. SJ: No. of swollen joints. TJ: No. of tender joints. Treat: psoriatic disease-related treatment. (-) no comorbidities present.
5

## Slide 6
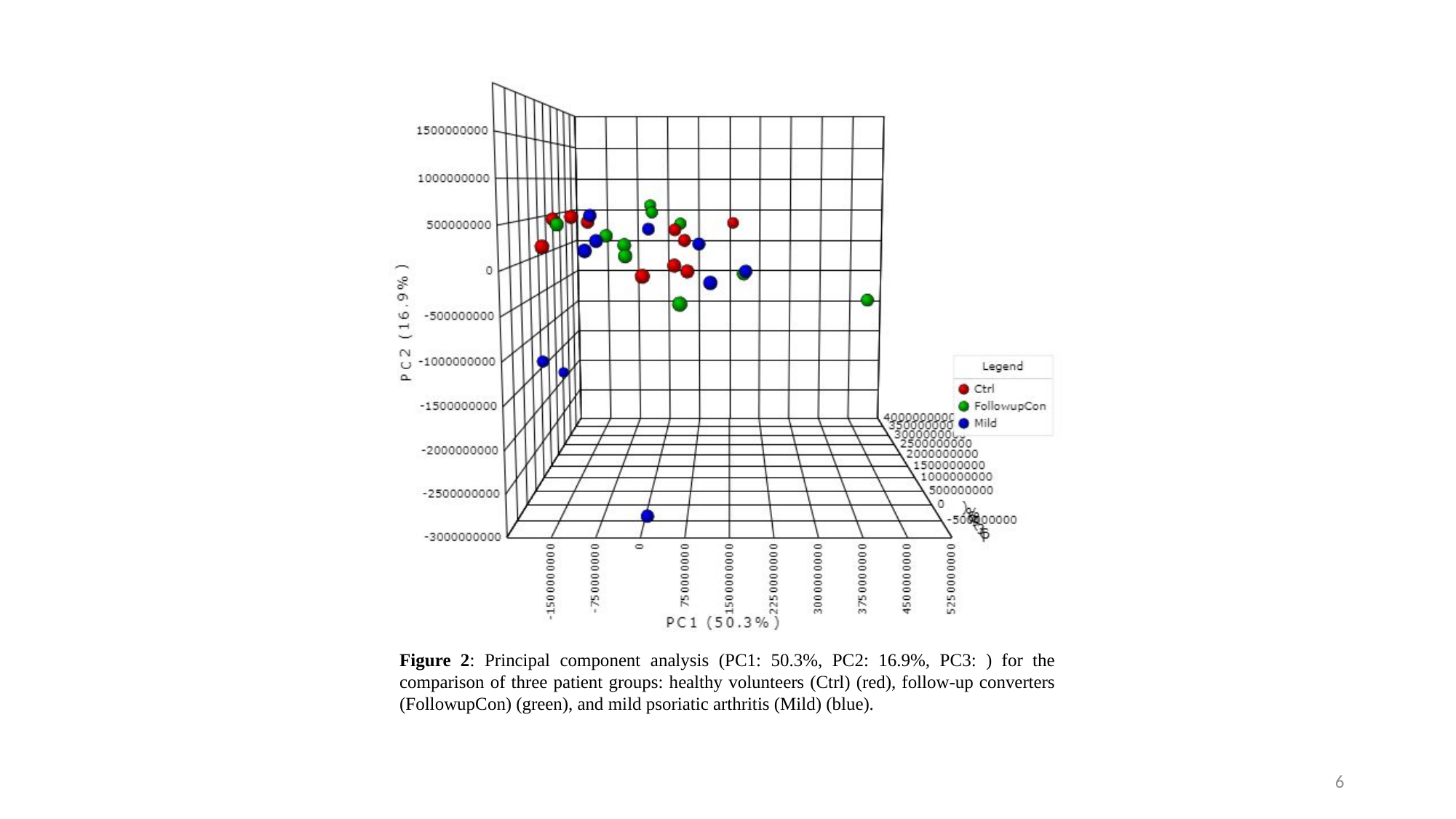

Figure 2: Principal component analysis (PC1: 50.3%, PC2: 16.9%, PC3: ) for the comparison of three patient groups: healthy volunteers (Ctrl) (red), follow-up converters (FollowupCon) (green), and mild psoriatic arthritis (Mild) (blue).
6

## Slide 7
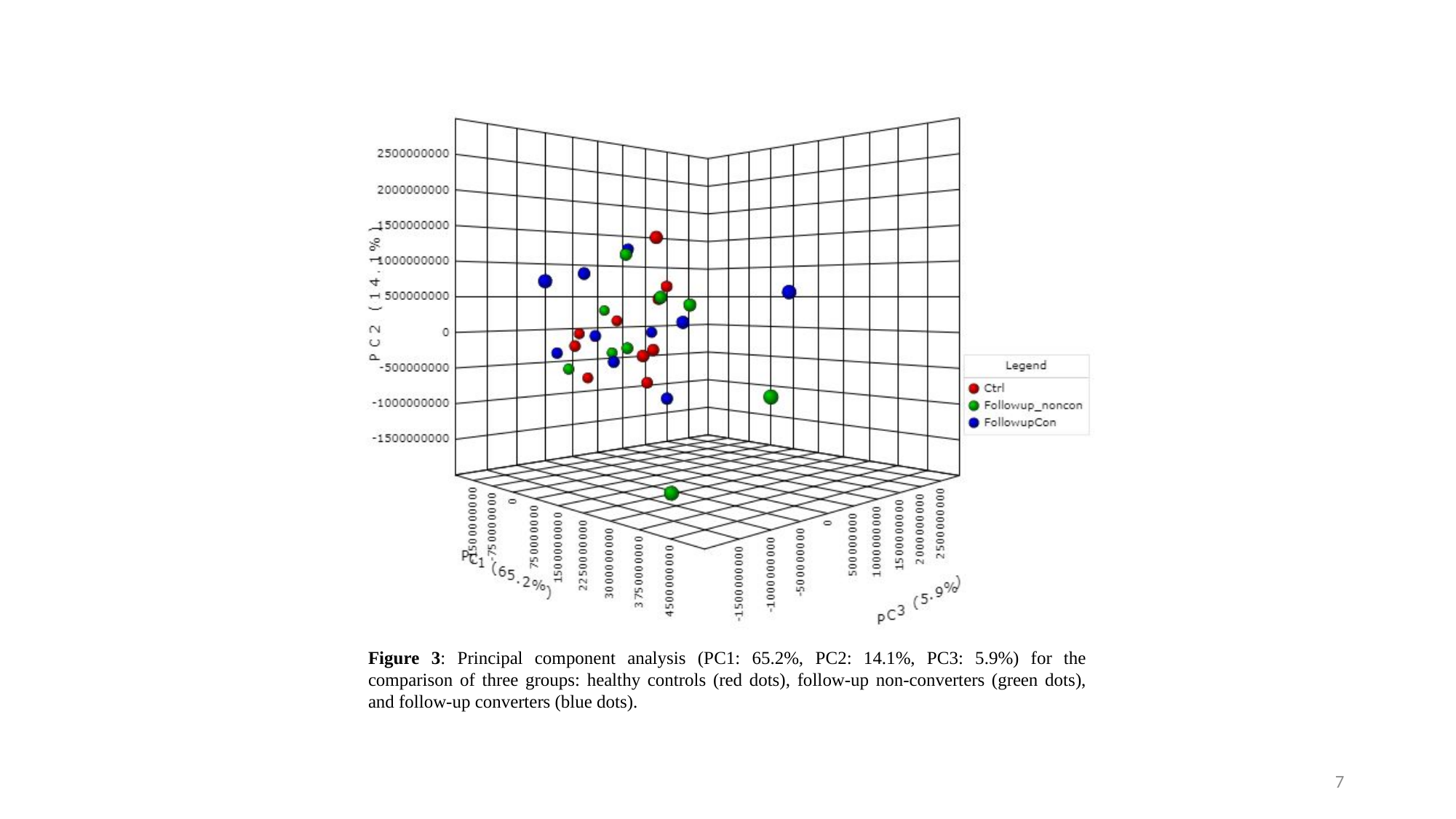

Figure 3: Principal component analysis (PC1: 65.2%, PC2: 14.1%, PC3: 5.9%) for the comparison of three groups: healthy controls (red dots), follow-up non-converters (green dots), and follow-up converters (blue dots).
7

## Slide 8
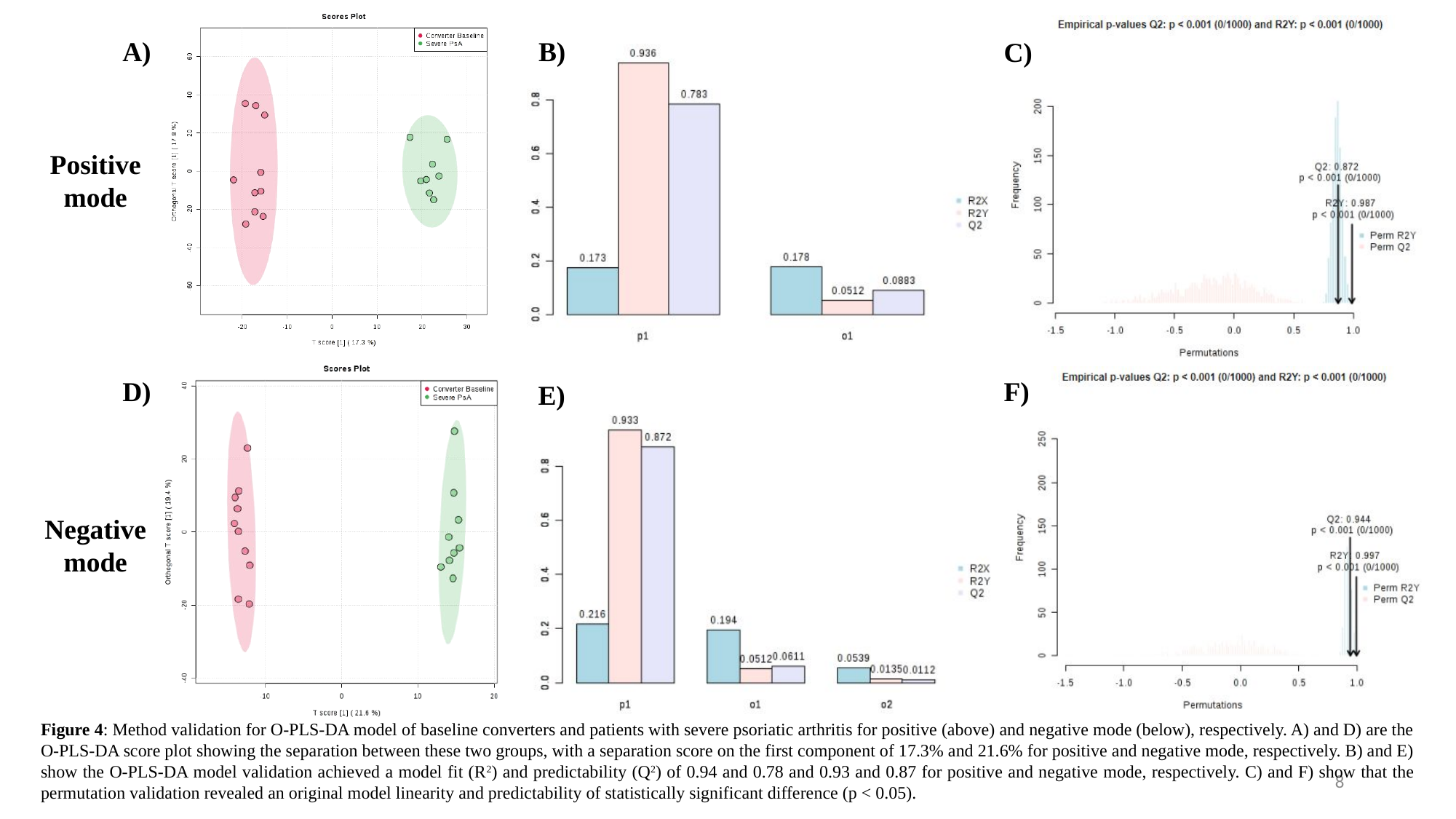

B)
A)
C)
Positive mode
D)
F)
E)
Negative mode
Figure 4: Method validation for O-PLS-DA model of baseline converters and patients with severe psoriatic arthritis for positive (above) and negative mode (below), respectively. A) and D) are the O-PLS-DA score plot showing the separation between these two groups, with a separation score on the first component of 17.3% and 21.6% for positive and negative mode, respectively. B) and E) show the O-PLS-DA model validation achieved a model fit (R2) and predictability (Q2) of 0.94 and 0.78 and 0.93 and 0.87 for positive and negative mode, respectively. C) and F) show that the permutation validation revealed an original model linearity and predictability of statistically significant difference (p < 0.05).
8

## Slide 9
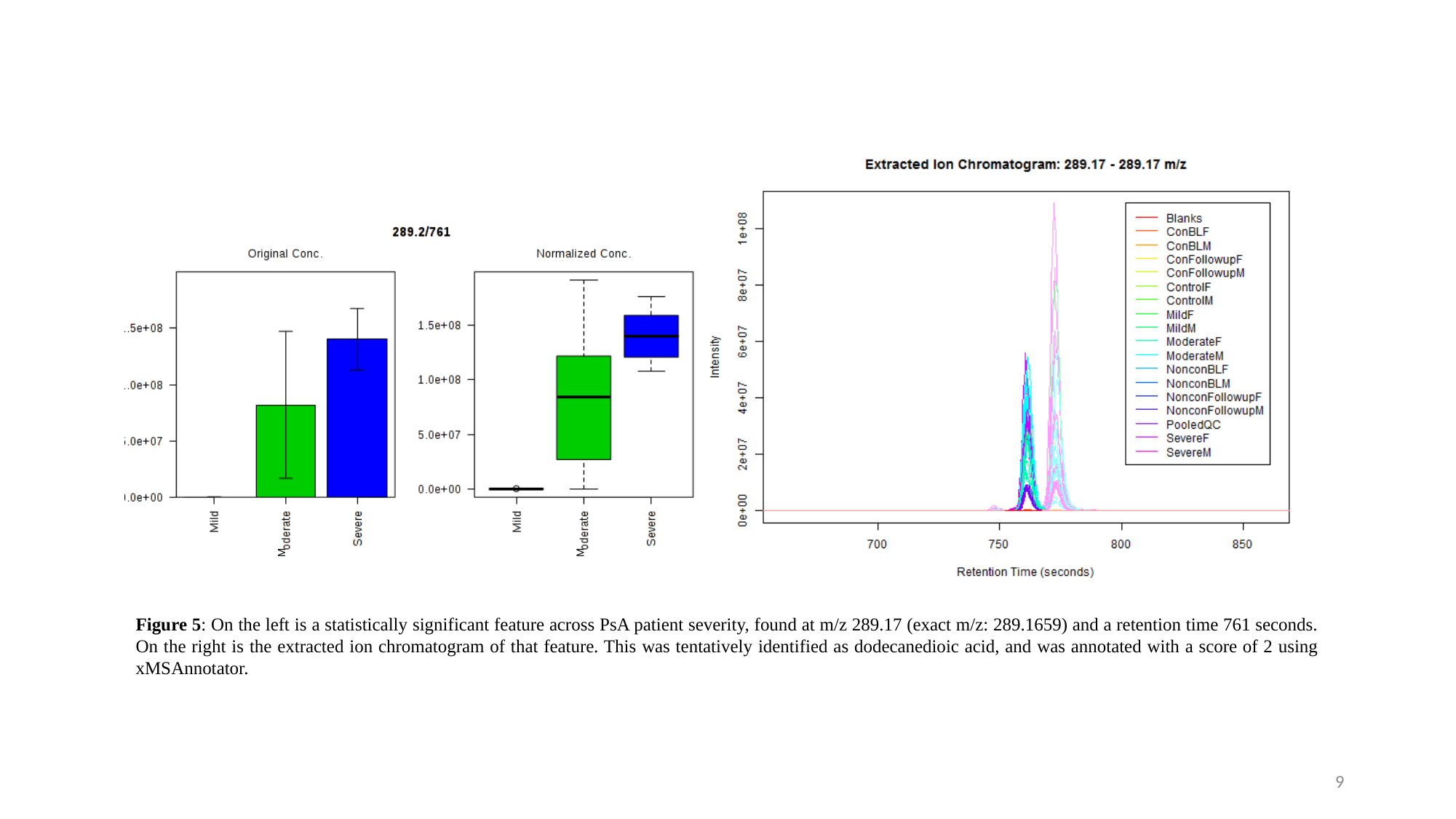

M
M
Figure 5: On the left is a statistically significant feature across PsA patient severity, found at m/z 289.17 (exact m/z: 289.1659) and a retention time 761 seconds. On the right is the extracted ion chromatogram of that feature. This was tentatively identified as dodecanedioic acid, and was annotated with a score of 2 using xMSAnnotator.
9

## Slide 10
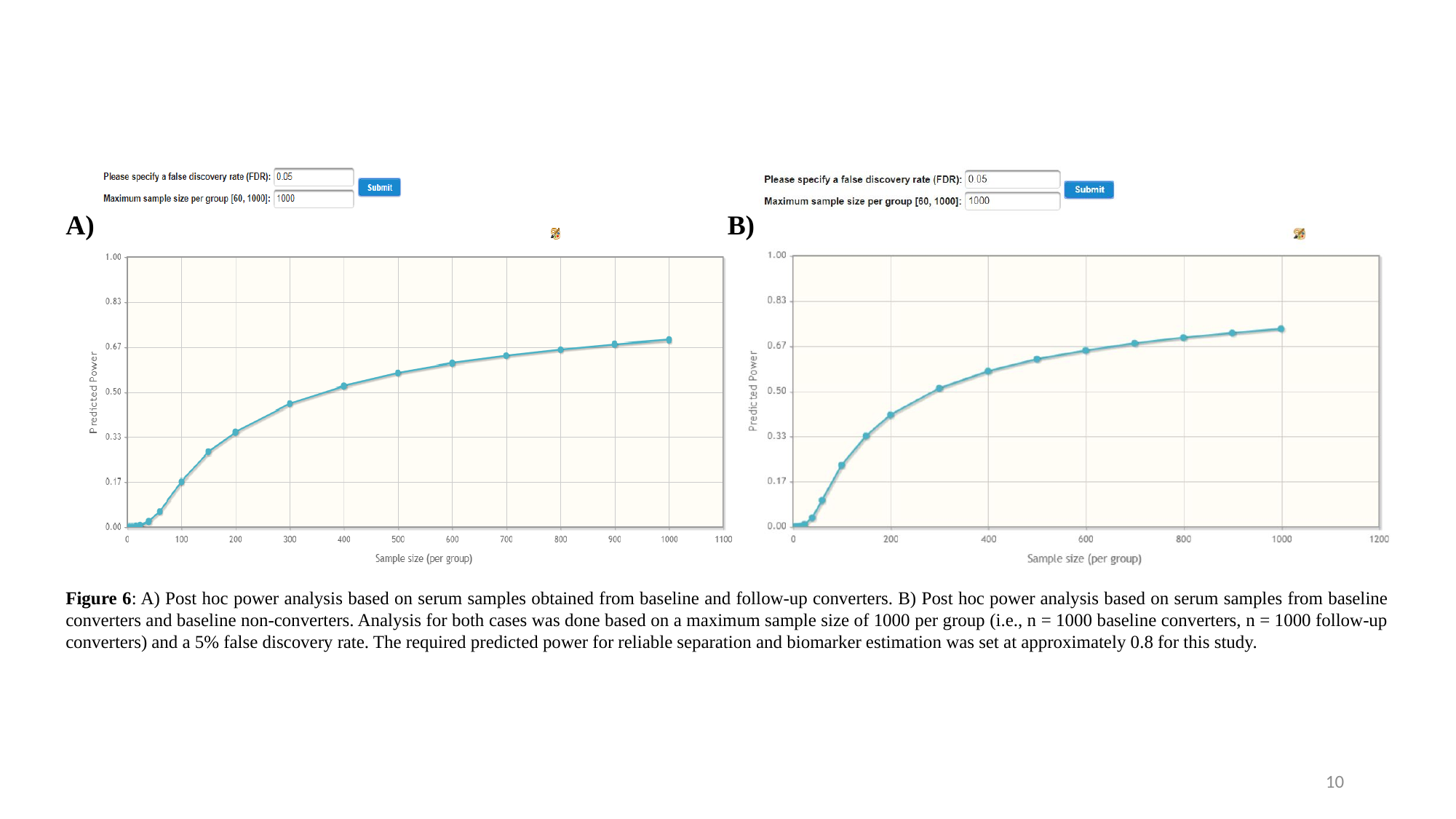

A)
B)
Figure 6: A) Post hoc power analysis based on serum samples obtained from baseline and follow-up converters. B) Post hoc power analysis based on serum samples from baseline converters and baseline non-converters. Analysis for both cases was done based on a maximum sample size of 1000 per group (i.e., n = 1000 baseline converters, n = 1000 follow-up converters) and a 5% false discovery rate. The required predicted power for reliable separation and biomarker estimation was set at approximately 0.8 for this study.
10

## Slide 11
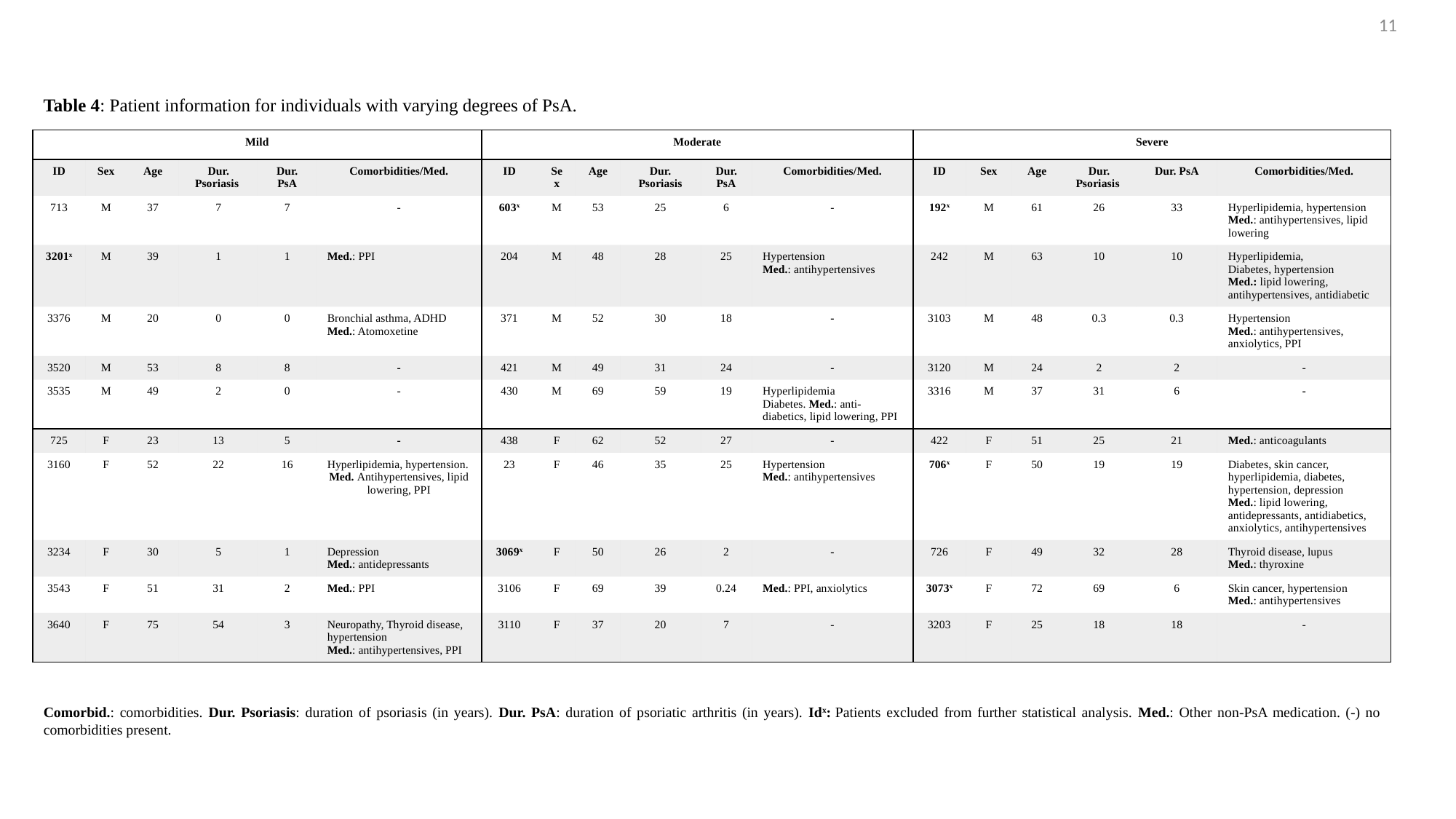

11
Table 4: Patient information for individuals with varying degrees of PsA.
| Mild | | | | | | Moderate | | | | | | Severe | | | | | |
| --- | --- | --- | --- | --- | --- | --- | --- | --- | --- | --- | --- | --- | --- | --- | --- | --- | --- |
| ID | Sex | Age | Dur. Psoriasis | Dur. PsA | Comorbidities/Med. | ID | Sex | Age | Dur. Psoriasis | Dur. PsA | Comorbidities/Med. | ID | Sex | Age | Dur. Psoriasis | Dur. PsA | Comorbidities/Med. |
| 713 | M | 37 | 7 | 7 | - | 603x | M | 53 | 25 | 6 | - | 192x | M | 61 | 26 | 33 | Hyperlipidemia, hypertension Med.: antihypertensives, lipid lowering |
| 3201x | M | 39 | 1 | 1 | Med.: PPI | 204 | M | 48 | 28 | 25 | Hypertension Med.: antihypertensives | 242 | M | 63 | 10 | 10 | Hyperlipidemia, Diabetes, hypertension Med.: lipid lowering, antihypertensives, antidiabetic |
| 3376 | M | 20 | 0 | 0 | Bronchial asthma, ADHD Med.: Atomoxetine | 371 | M | 52 | 30 | 18 | - | 3103 | M | 48 | 0.3 | 0.3 | Hypertension Med.: antihypertensives, anxiolytics, PPI |
| 3520 | M | 53 | 8 | 8 | - | 421 | M | 49 | 31 | 24 | - | 3120 | M | 24 | 2 | 2 | - |
| 3535 | M | 49 | 2 | 0 | - | 430 | M | 69 | 59 | 19 | Hyperlipidemia Diabetes. Med.: anti-diabetics, lipid lowering, PPI | 3316 | M | 37 | 31 | 6 | - |
| 725 | F | 23 | 13 | 5 | - | 438 | F | 62 | 52 | 27 | - | 422 | F | 51 | 25 | 21 | Med.: anticoagulants |
| 3160 | F | 52 | 22 | 16 | Hyperlipidemia, hypertension. Med. Antihypertensives, lipid lowering, PPI | 23 | F | 46 | 35 | 25 | Hypertension Med.: antihypertensives | 706x | F | 50 | 19 | 19 | Diabetes, skin cancer, hyperlipidemia, diabetes, hypertension, depression Med.: lipid lowering, antidepressants, antidiabetics, anxiolytics, antihypertensives |
| 3234 | F | 30 | 5 | 1 | Depression Med.: antidepressants | 3069x | F | 50 | 26 | 2 | - | 726 | F | 49 | 32 | 28 | Thyroid disease, lupus Med.: thyroxine |
| 3543 | F | 51 | 31 | 2 | Med.: PPI | 3106 | F | 69 | 39 | 0.24 | Med.: PPI, anxiolytics | 3073x | F | 72 | 69 | 6 | Skin cancer, hypertension Med.: antihypertensives |
| 3640 | F | 75 | 54 | 3 | Neuropathy, Thyroid disease, hypertension Med.: antihypertensives, PPI | 3110 | F | 37 | 20 | 7 | - | 3203 | F | 25 | 18 | 18 | - |
Comorbid.: comorbidities. Dur. Psoriasis: duration of psoriasis (in years). Dur. PsA: duration of psoriatic arthritis (in years). Idx: Patients excluded from further statistical analysis. Med.: Other non-PsA medication. (-) no comorbidities present.

## Slide 12
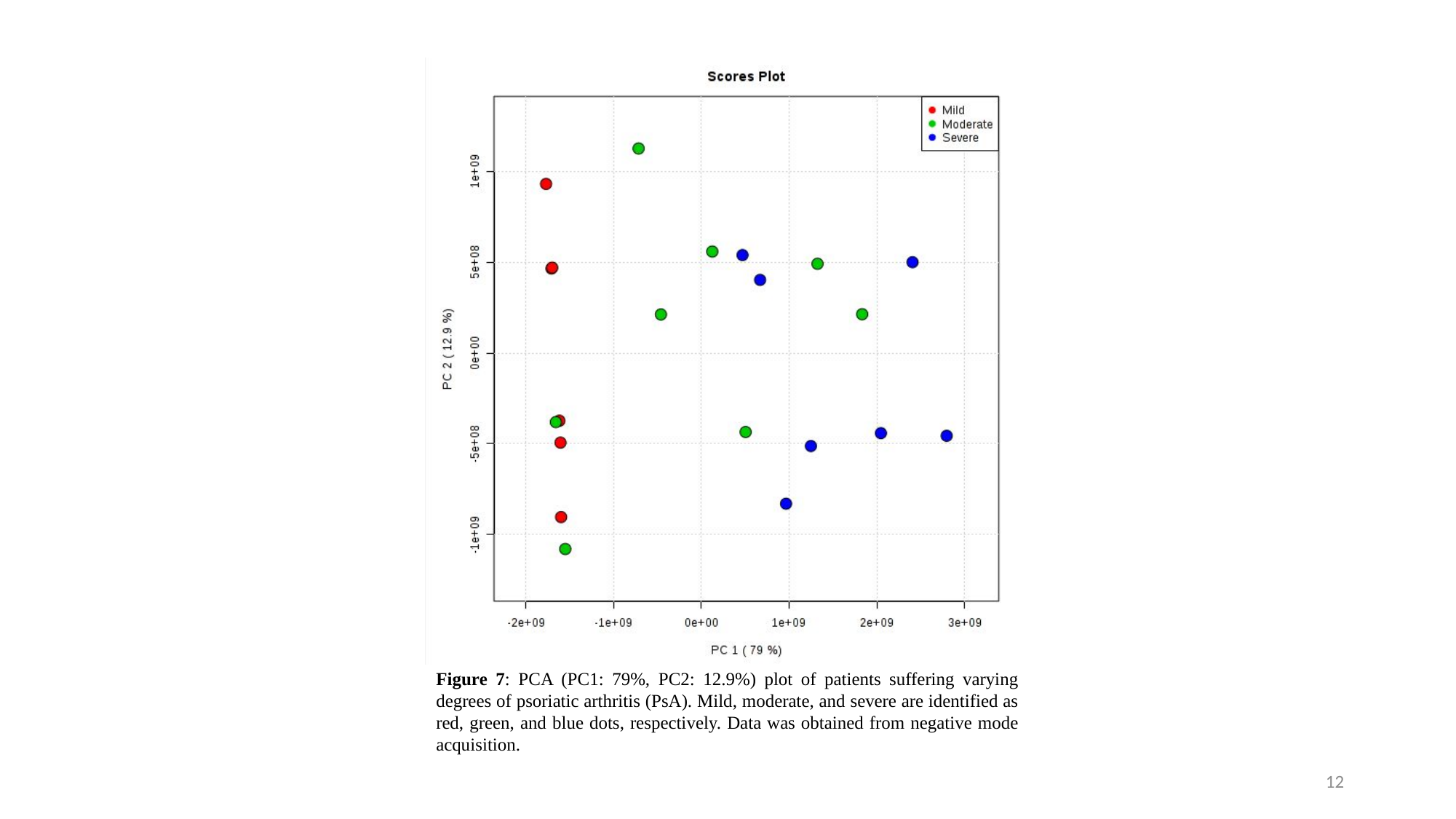

Figure 7: PCA (PC1: 79%, PC2: 12.9%) plot of patients suffering varying degrees of psoriatic arthritis (PsA). Mild, moderate, and severe are identified as red, green, and blue dots, respectively. Data was obtained from negative mode acquisition.
12
