# Supplementary material for: Serum metabolic fingerprinting of psoriasis and psoriatic arthritis patients using solid-phase microextraction—liquid chromatography—high-resolution mass spectrometry
Source: Metabolomics. 2021 Jun 16;17(7):59. doi: 10.1007/s11306-021-01805-3 (PMC8211611; doi:10.1007/s11306-021-01805-3)
Supplement: Supplementary file 2 — Supplementary file2 (DOCX 29 kb) [file 11306_2021_1805_MOESM2_ESM.docx]

**Online Resource 2**

**Serum metabolic fingerprinting of psoriasis and psoriatic arthritis patients using solid phase microextraction – liquid chromatography-high resolution mass spectrometry**

Nikita Looby^a^, Anna Roszkowska^a,b^, Nathaly Reyes-Garces^a^, Miao Yu^a^, Tomasz Bączek^b^, Vathany Kulasingam^c,d^*, Janusz Pawliszyn^a^*, Vinod Chandran^c,e,f,g^*

^a^ Department of Chemistry, University of Waterloo, 200 University Avenue, Waterloo, ON, Canada N2L 3G1

^b^ Department of Pharmaceutical Chemistry, Medical University of Gdańsk, Gdańsk, Poland

^c^ Department of Laboratory Medicine and Pathobiology, University of Toronto, Toronto, Canada

^d^ Division of Clinical Biochemistry, University Health Network, Toronto, Canada

^e^ Department of Medicine, Division of Rheumatology, University of Toronto, Toronto, Canada

^f^ Institute of Medical Science, University of Toronto, Toronto, Canada

^g^Schroeder Arthritis Institute, Krembil Research Institute, University Healthy Network, Toronto, ON, Canada, MT5 2S8

*Corresponding authors: Vathany Kulasingam, Ph.D., FCACB – Dr.[Vathany.Kulasingam@uhn.ca](mailto:Vathany.Kulasingam@uhn.ca); Janusz Pawliszyn, Ph.D., FCIC, FRSC - [janusz@uwaterloo.ca](mailto:janusz@uwaterloo.ca); Vinod Chandran, MB., BS., MD., DM., Ph.D. - [Vinod.Chandran@uhnresearch.ca](mailto:Vinod.Chandran@uhnresearch.ca)

**2.2.1 Materials**

Thin-film stainless steel combs were purchased from PAS technologies (Magdala, Germany), while oasis hydrophilic-lipophilic balanced (HLB) particles (30 – 60 µm) and weak anion exchanger functionalized polystyrene divinylbenzene (PS-DVB-WAX) particles (30 – 60 µm) were purchased from Waters Corporation (Milford, USA) and Chromatographic Specialties, respectively (Brockville, Canada). A flask-type sprayer and the following internal standards and chemicals were purchased from Millipore Sigma (Burlington, USA): tranexamic acid, phenylalanine-d5, testosterone-d3, codeine-d3, sodium chloride, potassium chloride, sodium phosphate, potassium phosphate, polyacrylonitrile, and N,N-dimethylformamide (DMF). 2 mL deep-well plates were purchased from Thermo Fisher Scientific (Waltham, USA), and the following LC-MS grade solvents were purchased from Fisher Scientific (Ottawa, Canada): formic acid, acetic acid, acetonitrile, methanol, and water. The deionized water that was used to prepare the phosphate buffered saline was obtained using an Milli-Q Reference Water Purification System (EMD Millipore, Fisher Scientific, Ottawa, Canada).

**2.2.2 Thin-film microextraction device preparation**

The thin-film microextraction (TFME) device was prepared using a well-established spraying method that had been developed in-laboratory (Mirnaghi et al. 2011). Briefly, a flask-type sprayer was used to coat the stainless-steel support with a slurry mixture consisting of 1:1 HLB and PS-DVB-WAX particles, polyacrylonitrile (to act as a binder), and DMF. After the application of each layer, the TFME devices were placed in an oven to cure at 150 °C for 1 minute. The final coating (extraction phase) on the device was 1 cm long with an average thickness of 0.35 ± 0.03 mm.

**2.2.3 Sample Collection**

The patient samples are collected in clinic soon after clinical assessment and in accordance with standard operating procedures and protocols. Blood samples were collected in red-top tubes and the clotting process lasted at least 20-30 minutes. After centrifugation, serum is collected, aliquoted, appropriately labelled, dated and stored at -80°C until time of analysis.

**2.2.4 Sample preparation**

Serum samples were processed using TFME, which consisted of a thin stainless-steel blade that had been coated with an appropriate sorbent (see section 2.3). Each well of the 96-well plate were filled with 200 µL of serum and 400 µL of phosphate buffered saline (PBS) containing deuterated internal standards at a final concentration of 50 µg/mL (unless otherwise stated) including: eicosapentonaoic acid-d5 (at 200 µg/mL), phenylalanine-d5, testosterone-d3, oxycodone-d3, codeine-d3, diazepam-d5 and warfarin-d5. Thus, each well contained a final sample volume of 600 µL. The well was then mildly agitated (300-500 rpm) for homogeneity after dilution and to establish protein binding prior to sample preparation with TFME. The coating (extraction phase) was first conditioned in a mixture of 1:1 methanol:water (v/v) for 30 minutes at 1500 rpm, while the serum samples were incubated at 25 °C during this time. Following conditioning, the TFME device was rinsed in water for 30 seconds at 1500 rpm and then immersed into serum samples for the extraction of metabolites for a period of 1 hour at 1500 rpm. After the extraction process, the device was rinsed in water for 10 seconds at 1500 rpm to remove any loosely attached matrix components from its surface. Finally, the extracted metabolites were desorbed in 550 µL of a mixture consisting of 4:3:3 methanol: acetonitrile:water (v/v/v) for 1 hour at 1500 rpm. The use of a Concept-96 autosampler to automate the TFME protocol enabled a sample preparation time of under 2 minutes per sample. The desorption solution was then diluted with 220 µL of water to produce a final extract composed of 1:1 organic/aqueous content, thus ensuring compatibility with the initial chromatographic conditions of the LC-HRMS metabolomics method that would be used for analysis. A pooled quality control (QC) sample was prepared by combining 10 µL of each sample extract (not including randomly chosen sample replicates) in a separate well. The pooled QC was injected approximately every 10 sample injections during instrumental analysis.

**2.2.5 Instrumental analysis: liquid chromatography coupled with high-resolution mass spectrometry**

High performance liquid chromatography (HPLC) with HRMS detection was performed using an Accela autosampler and pump coupled to a Q-Exactive mass spectrometer (Thermo Fisher Scientific, Waltham, USA). Chromatographic separation was conducted on a Discovery HS F5-3 column (100 mm x 2.1 mm, 3 µm) with a Discovery HS F5 (20 mm x 2.1mm, 3 µm) pre-column, which were both purchased from Supelco/Sigma Aldrich (Bellefonte, USA). Gradient elution was achieved over 40 minutes in positive mode using mobile phases consisting of 99.9/0.1 water/formic acid (v/v) and 99.9/0.1 acetonitrile/formic acid (v/v). For negative-mode chromatography, 1mM of acetic acid was used instead of formic acid. Sample extracts were injected at a volume of 10 µL, and the autosampler and column temperature were maintained at 5 °C and 25 °C, respectively. Further details on the gradient elution used for chromatography can be found in the table below.

| **MS polarity** | **Positive mode** | **Negative mode** |
| --- | --- | --- |
| **Time (min)** | **% Mobile phase A (water + 0.1 % formic acid** | **% Mobile phase A (water + 1mM acetic acid** |
| 0.00 | 100 | 100 |
| 3.00 | 100 | 100 |
| 25.00 | 10 | 10 |
| 34.00 | 10 | 10 |
| 35.00 | 100 | 100 |
| 40.00 | 100 | 100 |

The Q-Exactive mass spectrometer was equipped with an Ion Max heating source, which contained a heated electrospray ionization (HESI-II) probe. The mass spectrometer was run at high resolution (50, 000), and data was acquired within an m/z range of 100 – 1000 with a balanced automatic gain control and an injection time of 100 milliseconds. The capillary and vaporizer temperatures were each set at 300 °C, while the sheath, auxiliary, and sweep gas were set to 35, 5, and 0, respectively. The electrospray voltages (and lock masses) applied for positive and negative mode were 4 kV (391.2842 m/z) and -2.9 kV (255.2329 m/z), respectively.

**2.2.6 Data pre-treatment and analysis**

The raw LC-MS data files acquired during instrumental analysis were first converted to mzXML files using MSConvert (Chambers et al. 2012). The converted files were then pre-processed using the XCMS software package and an RStudio script that had been developed in-laboratory; pre-processing involved performing grouping, retention time correction, peak alignment, peak filling, peak picking etc. (Roszkowska et al. 2018; Smith et al. 2006). Optimized parameters for data pre-processing were obtained using the IPO package (Libiseller et al. 2015) on selected pooled QC samples run at the beginning, middle, and end of the sequence, while the extracted peaks were annotated using the xMSAnnotator Integrative scoring algorithm (Uppal et al. 2017) in conjunction with the Human Metabolome Database (HMDB) (Wishart et al. 2007). More detailed statistical analysis was achieved using Metaboanalyst [accessed September 5, 2017] (Chong et al. 2018), and XCMS online (Huan et al. 2017) and METLIN (Smith et al. 2005) were also utilized to perform the initial data analysis and feature identification, respectively, on the preliminary results.

The peak-list generated using the XCMS software package in RStudio were initially filtered using the relative standard deviation (RSD) of the pooled QCs, with any feature in the pooled QC with > 30 % RSD being removed from further analysis. Secondary filtering methods were conducted based on solvent and device blanks, with any feature with a pooled-QC:device/solvent-blank peak-intensity ratio of < 5 also being removed from further statistical evaluation. The resultant peak-list was uploaded to Metaboanalyst; for this web-based platform, the default settings were used for missing values, and no additional feature filtering was applied. In addition, no further data normalization, data transformation, nor scaling (no differences were observed between the PCA’s generated for no scaling, autoscaling, or pareto scaling) were performed prior to univariate or multivariate chemometric analyses. Since the distribution of data cannot be assumed, a Kruskal Wallis Test and/or a Wilcoxon rank test (performed when necessary) with a false discovery rate (FDR) adjusted p-value of 0.05 was applied for univariate analysis. Prior to that test, the possibility to perform parametric test was verified, however, one or more assumptions were not fulfilled: normal distribution – checked with Shapiro-Wilk test and Kolmogorov-Smirnov test, homoscedasticity (homogeneity of variance) – checked with Levene’s test and Brown-Forsythe test and the correlation of means and variances. For multivariate analysis, Principal Component Analysis (PCA), Partial Least Squares–Discriminant Analysis (PLS-DA), and Orthogonal Projection to Latent Structures-Discriminant Analysis (OPLS-DA) were performed, as necessary. Given the small number of patients in each group (n ≤ 10 if there are outliers), model validation for the supervised multivariate analyses (O-PLS-DA and PLS-DA) was conducted using a combination of permutation (at 1000) and leave-one-out cross-validation (LOOCV). Successful model validation for O-PLS-DA was achieved when the original model passed permutation at p < 0.05 for model predictability (Q^2^) and/or model fit (R^2^). For a PLS-DA model, successful model validation was achieved with a permutation (for separation distance) of p < 0.05 and when Q^2^ and R^2^ > 0.6 and/or were within 0.2 units of each other. Only features with a Variable Importance in Projection (VIP) score of > 1 were investigated further.
