# Supplementary material for: Serum metabolic fingerprinting of psoriasis and psoriatic arthritis patients using solid-phase microextraction—liquid chromatography—high-resolution mass spectrometry
Source: Metabolomics. 2021 Jun 16;17(7):59. doi: 10.1007/s11306-021-01805-3 (PMC8211611; doi:10.1007/s11306-021-01805-3)
Supplement: Supplementary file 3 — Supplementary file3 (DOCX 79 kb) [file 11306_2021_1805_MOESM3_ESM.docx]

**Online Resource 3**

**Serum metabolic fingerprinting of psoriasis and psoriatic arthritis patients using solid phase microextraction – liquid chromatography-high resolution mass spectrometry**

Nikita Looby^a^, Anna Roszkowska^a,b^, Nathaly Reyes-Garces^a^, Miao Yu^a^, Tomasz Bączek^b^, Vathany Kulasingam^c,d^*, Janusz Pawliszyn^a^*, Vinod Chandran^c,e,f,g^*

^a^ Department of Chemistry, University of Waterloo, 200 University Avenue, Waterloo, ON, Canada N2L 3G1

^b^ Department of Pharmaceutical Chemistry, Medical University of Gdańsk, Gdańsk, Poland

^c^ Department of Laboratory Medicine and Pathobiology, University of Toronto, Toronto, Canada

^d^ Division of Clinical Biochemistry, University Health Network, Toronto, Canada

^e^ Department of Medicine, Division of Rheumatology, University of Toronto, Toronto, Canada

^f^ Institute of Medical Science, University of Toronto, Toronto, Canada

^g^ Schroeder Arthritis Institute, Krembil Research Institute, University Healthy Network, Toronto, ON, Canada, MT5 2S8

*Corresponding authors: Vathany Kulasingam, Ph.D., FCACB – Dr.[Vathany.Kulasingam@uhn.ca](mailto:Vathany.Kulasingam@uhn.ca); Janusz Pawliszyn, Ph.D., FCIC, FRSC - [janusz@uwaterloo.ca](mailto:janusz@uwaterloo.ca); Vinod Chandran, MB., BS., MD., DM., Ph.D. - [Vinod.Chandran@uhnresearch.ca](mailto:Vinod.Chandran@uhnresearch.ca)

**S1:** The most distinctive differences for 3-hydroxytetradecanedioic acid (identified with m/z value of 275.1854) were noted for patients with moderate and severe PsA. The comparisons of severe and moderate PsA with healthy controls brought similar relations (p < 1x10-3; Kruskal-Wallis test for severe PsA vs. Control and p < 1,5x10-2; Kruskal-Wallis test for Moderate PsA vs. Control). The signal for 3-hydroxytetradecanedioic acid was also identified based on its m/z value (297.1673). For that identification statistically significant differences in the following pairs of groups could be distinguished: baseline converters vs. moderate PsA, baseline converters vs. severe PsA, follow-up converters vs. moderate PsA, follow-up converters vs. severe PsA, heathy controls vs. moderate PsA, healthy controls vs. severe PsA, moderate PsA vs. baseline non-converters, moderate PsA vs. follow-up_non-converters, severe PsA vs. baseline non-converters, severe PsA vs. follow-up_non-converters. Similarly, we found several statistically significant relationships for L-phenylalanine with moderate PsA differing significantly (p < 0.05; Kruskal-Wallis test) with baseline converters, follow-up converters, and healthy controls. The same relationships were also noted for severe PsA. Indeed, all putatively identified compounds were found to increase in relation to disease activity, with mild PsA exhibiting the lowest levels, moderate PsA exhibiting much higher levels, and severe PsA exhibiting the highest levels between the three groups. An example of this trend is demonstrated in Online Resource 1 – Figure 5 specifically for dodecanedioic acid.

**Table 1** Tentatively annotated features showing statistically significant differences via univariate analysis across patients with varying severity of psoriatic arthritis disease activity for negative mode data.

| **Feature No.** | **Tentative ID** | **m/z** | **Adduct** | **Retention time (min)** | **P value** | **Q value** | **Annotation score (max = 3)** |
| --- | --- | --- | --- | --- | --- | --- | --- |
| 4448 | 2-Hydroxydecanedioic acid, 3-Hydroxysebacic acid  cis-4-Hydroxycyclohexylacetic acid | 217.1077 | [M-H]^-^    [M+CH3COO]^-^ | 11.78 | 1.5x10^-3^ | 3.0x10^-2^ | 2 |
| 3752 | p-Coumaroylagmatine  Undecanedioic acid | 275.1501 | [M-H]^-^  [M+CH3COO]^-^ | 12.15 | 8.5x10^-4^ | 3.0x10^-2^ | 2 |
| 1139 | 3-Hydroxydodecanedioic acid | 281.1162 | [M+Cl]^-^ | 14.00 | 1.9x10^-3^ | 3.0x10^-2^ | 2 |
| 3689 | Dodecanedioic acid | 289.1659 | [M+CH3COO]^-^ | 12.68 | 2.2x10^-3^ | 3.0x10^-2^ | 2 |
| 216 | S-aminomethyldihydrolipoamide | 295.1163 | [M+CH3COO]^-^ | 13.36 | 9.3x10^-4^ | 3.0x10^-2^ | 0 |
| 2254 | 1,11-Undecanedicarboxylic acid | 303.1818 | [M+CH3COO]^-^ | 13.40 | 6.4x10^-3^ | 4.0x10^-2^ | 2 |
| 2412 | Phenylbutyrylglutamine | 327.1118 | [M+Cl]^-^ | 13.03 | 1.9x10^-3^ | 3.0x10^-2^ | 2 |
| 2240 | Arginyl-Lysine | 337.1744 | [M+Cl]^-^ | 12.30 | 1.4x10^-3^ | 3.0x10^-2^ | 2 |
| 2522 | 6-Keto-PGF1a | 429.2488 | [M+CH3COO]^-^ | 18.90 | 1.5x10^-3^ | 3.0x10^-2^ | 2 |
| 2256 | N1-(alpha-D-ribosyl)-5,6-dimethyl-benzimidazole  L-phenylalanyl-l-hydroxyproline  Prolyl-Tyrosine | 313.0959 | [M+Cl]^-^  [M+Cl]^-^  [M+Cl]^-^ | 12.25 | 1.5x10^-3^ | 3.0x10^-2^ | 2 |

**Table 2** Tentatively annotated features showing statistically significant differences via univariate analysis between healthy controls and severe psoriatic arthritis patients for positive mode data.

| **Feature No.** | **Tentative ID** | **m/z** | **Adduct** | **Retention time (min)** | **P value** | **Q value** | **Annotation score (max = 3)** |
| --- | --- | --- | --- | --- | --- | --- | --- |
| 57 | S-aminomethyldihydrolipoamide | 237.1090 | [M+H]^+^ | 13.25 | 8.0x10^-5^ | 1.8x10^-2^ | 2 |
|  |  |  |  |  |  |  |  |
| 130 | Hydroxycapric acid | 189.1485 | [M+H]^+^ | 14.84 | 2.0x10^-4^ | 2.0x10^-2^ | 3 |
| 304 | Glutamyl-tyrosine | 310.1159 | [M+H]^+^ | 14.01 | 5.7x10^-4^ | 4.3x10^-2^ | 2 |
| 365 | Methyladenosine  Isoleucyl glutamate | 282.1197  282.1186 | [M+H]^+^   [M+Na]^+^ | 12.17 | 7.0x10^-4^ | 2.6x10^-2^ | 2 |
| 531/1302 | Adenosine or deoxyguanosine  Isobutyryl carnitine  S-aminomethyldihydrolipoamide | 285.1306   254.1363   254.1355 | [M+NH4]^+^   [M+Na]^+^  [M+NH4]^+^ | 11.05 | 1.0x10^-3^ | 3.0x10^-2^ | 0 |
| 549 | Gamma-glutamyltyrosine | 311.1238 | [M+H]^+^ | 13.45 | 1.0x10^-3^ | 3.0x10^-2^ | 2 |
| 715 | Arginyl-glycine | 254.1224 | [M+Na]^+^ | 13.33 | 2.0x10^-3^ | 4.0x10^-2^ | 1 |

**Table 3** Variables of importance isolated from validated O-PLS-DA model that differentiate baseline converters from severe psoriatic arthritis. Data obtained from positive mode acquisition.

| **Feature No.** | **Tentative ID** | **m/z** | **Adduct** | **Retention time (min)** | **VIP score** | **Biochemical importance** |
| --- | --- | --- | --- | --- | --- | --- |
| 8728 | 3-Hydroxytetradecanedioic acid | 275.1854 | [M+H]^+^ | 15.72 | 30 | High levels of 3-Hydroxytetradecanedioic acid (and other 3-hydroxydicarboxylic acids) were detected in the urine of a patient with 3-hydroxydicarboxylic aciduria. |
| 8834 | 3-Hydroxytetradecanedioic acid | 297.1673 | [M+Na]^+^ | 15.70 | 24 |  |
| 3800 | L-phenylalanine, 3-pyridinebutanoic acid  Pyruvophenone, dihydrocoumarin | 166.0865 | [M+H]^+^  [M+NH4]^+^ | 10.85 | 15 | When present in sufficiently high levels, phenylalanine can act as a neurotoxin and a metabotoxin. A metabotoxin is an endogenously produced metabolite that causes adverse health effects at chronically high levels. Chronically high levels of phenylalanine are associated with at least five inborn errors of metabolism. |
| 9905 | 9,12-Dioxo-dodecanoic acid | 229.1437 | [M+H]^+^ | 13.8 | 8 | Di-oxo-dicarboxylic acid. |
| 10122 | Deoxyadenosine  3-Hydroxydodecanedioic acid | 269.1361 | [M+NH4]^+^  [M+Na]^+^ | 13.8 | 8 | When present in sufficiently high levels, deoxyadenosine can act as an immunotoxin and a metabotoxin. Chronically high levels of deoxyadenosine are associated with adenosine deaminase (ADA) deficiency, an inborn error of metabolism. |
| 9043 | 3-Hydroxydodecanedioic acid | 247.1542 | [M+H]^+^ | 13.8 | 7 | A dicarboxylic acid that appears in the urine of children affected with peroxisomal disorders. |
| 8393 | 2-Phenylglycine | 152.0709 | [M+H]^+^ | 9.1 | 7 | Has a role as a human metabolite and is described in normal human urine. |
| 8434 | Ribonic acid, lyxonate, apionic acid, arabinonic acid | 184.081 | [M+NH4]^+^ | 13.8 | 2 | Sugar carboxylic acids. |
| 10328 | Biopterin | 255.1204 | [M+NH4]^+^ | 12.9 | 2 | Biopterin concentrations in cerebrospinal fluid from patients with Parkinson's disease are lower than those from age-matched older controls. Lowered levels of urinary biopterin concomitant with elevated serum phenylalanine concentration occur in a variant type of hyperphenylalaninemia. |
| 2321 | Creatinine | 114.0666 | [M+H]^+^ | 3.7 | 1.4 | A breakdown product of creatine phosphate in muscle. |
| 2830 | L-tyrosine, hydroxy butanoic acid, amino(hydroxyphenyl)propanoate, hydroxyphenylalanine  M-coumaric acid, phenyl pyricin acid | 182.0814 | [M+H]^+^  [M+NH4]^+^ | 8.78 | 1.4 | Tyrosine is not found in large concentrations throughout the body, likely because it is rapidly metabolized.  Most common is the increased amount of tyrosine in the blood of premature infants, which is marked by decreased motor activity, lethargy, and poor feeding. Hydroxybutyric acid (also known as gamma-hydroxybutyrate or GHB) is a precursor and a metabolite of gamma-aminobutyric acid. Hydroxyphenylalanine is an L-phenylalanine derivative. |
| 8861 | 3-Hydroxytetradecanedioic acid | 292.212 | [M+NH4]^+^ | 15.71 | 1.2 | See feature No. |
| 1185 | Isoleucine | 132.1022 | [M+H]^+^ | 8.93 | 1.1 | Branched amino acids have different metabolic routes: leucine which is solely involved in fats; and isoleucine which is involved in both fats and carbohydrates. |
| 9590 | Indoleacetaldehyde | 177.1025 | [M+NH4]^+^ | 9.4 | 1 | Indoleacetaldehyde participates in a number of enzymatic reactions.  In particular, indoleacetaldehyde can be biosynthesized from tryptamine, which is mediated by the enzyme kynurenine 3-monooxygenase. Indoleacetaldehyde is involved in tryptophan metabolism. |

**Table 4** Variables of importance (VIPs) isolated from validated O-PLS-DA model that differentiate baseline converters from severe psoriatic arthritis. Data obtained from negative mode acquisition.

| **Feature No.** | **Tentative ID** | **m/z** | **Adduct** | **Retention time (min)** | **VIP score** | **Biochemical importance** |
| --- | --- | --- | --- | --- | --- | --- |
| 1638 | 3-hydroxydodecanedioic acid  10-hydroxy-2E-decenoic acid, 2-oxo capric acid | 245.1393 | [M-H]^-^  [M+CH3COO]^-^ | 14.00 | 27 | 3-hydroxydodecanedioic acid is a medium chain hydroxy fatty acid that reportedly appears in children affected with peroxisomal disorders. PMID: [10896310](https://www.ncbi.nlm.nih.gov/pubmed/10896310). |
| 77 | 12-amino-dodecanoic acid | 274.2027 | [M+CH3COO]^-^ | 15.93 | 6.8 | This is an omega-amino medium chain fatty acid and has a role as a bacterial metabolite. (PubChem). |
| 404 | n-heptanoyl acetic acid, 3-caproyl propionic acid, 7-methyl-3-oxooctanoic acid, 4-n-valeryl butyric acid | 150.0008 | [M+CH3COO]^-^ | 14.13 | 6.2 | Medium chain oxo-monocarboxylic acids. (PubChem). |
| 1151 | 2,4-Dideoxy-2-octylpentaric acid  2R-Hydroxy-10-undecenoic acid, 3-capryl propionic acid | 259.1552 | [M-H]^-^  [M+CH3COO]^-^ | 14.96 | 3.5 | Medium chain hydroxy and “ene”-monocarboxylic acids. |
| 2478 | 2-Hydroxydecanedioic acid, 3-hydroxy-sebacic acid, 2-hydroxy-decanedioic acid  3-Oxovalproic acid, 4-Hydroxycyclohexylacetic acid, 2-keto-n-caprylic acid | 217.1076 | [M-H]^-^  [M+CH3COO]^-^ | 12.25 | 2.6 | Medium chain hydroxy dicarboxylic acids. (PubChem) 3-Hydroxysebacic acid is a normal urinary metabolite and can be elevated in patients with peroxisomal disorders. 3-Hydroxysebacic acid and caprylic acid are found to be associated with medium chain acyl-CoA dehydrogenase deficiency, which are inborn errors of metabolism. 4-Hydroxycyclohexylacetic acid is a tyrosine metabolite that has been found in the urine of a patient with a defect of 4-hydroxyphenylpyruvate dioxygenase, an inborn error of metabolism. (HMDB). |
| 2692 | p-Cresol sulfate  Thiophenecarboxylic acid | 187.0062 | [M-H]^-^  [M+CH3COO]^-^ | 10.23 | 2.1 | p-Cresol sulfate is a microbial metabolite that is found in urine and likely derives from secondary metabolism of p-cresol. It appears to be elevated in the urine of individuals with progressive multiple sclerosis (HMDB). |
| 2891 | Androsterone sulfate | 369.1743 | [M-H]^-^ | 15.93 | 1.3 | Androsterone sulfate is clinically recognized as one of the major androgen metabolites found in urine. (HMDB). |
| 651 | 1,2-Dihexanoyl-sn-glycerol, 6-hydroxypentadecanedioic acid  Keto tridecanoic acid, hydroxy-9-tridecenoic acid, 7-methoxy-dodec-4-enoic acid, 6-hydroxy-4-tridecanolide | 287.1865 | [M-H]^-^  [M+CH3COO]^-^ | 16.85 | 1.1 | 1,2-Dihexanoyl-sn-glycerol is a hexanoic acid derivative. Majority of other tentative compounds are dicarboxylic and carboxylic acid derivatives. (PubChem). |
| 1282 | 2-Aminoadenosine | 341.1223 | [M+CH3COO]^-^ | 14.02 | 1.1 | Purine nucleoside. (PubChem). |

**Table 5** Unique (U) and multiple (M) features with 2 (medium) to 3 (high) confidence matches annotated by HMDB. The annotation was based on intensity profiles, retention time, mass defect, and isotope/adduct patterns of peaks. In high confidence match, non-zero xMSannotator multistage score, required adducts, N, O, P, S/C ratio check, hydrogen/carbon ratio check, abundance ratio checks for isotopes, multimers and multiply charged adducts are satisfied. In medium confidence match, pathway level correlation is satisfied.

| **Category** | **Compound(s)** | **m/z** | **Adduct(s)** | **Retention time (sec)** | **Confidence match** |
| --- | --- | --- | --- | --- | --- |
| LIPIDS | 6-hydroxypentadecanedioic acid | 311.1829  289.201 | M+Na  M+H | 997.1 | U3 |
|  | 3-hydroxytetradecanedioic acid | 275.1854 | M+H | 943.1 | U2 |
|  | 3-hydroxydodecanedioic acid | 269.1361  229.1437  247.1542  537.2651 | M+Na  M+H-H2O  M+H  2M+2Na-H | 829.1 | M3 |
|  |  | 246.1427  245.1393 | M-H_[-1]  M-H | 832 | M3 |
|  | sphingosine 1-phosphate (d16:1-P) | 352.2247 | M+H | 1058.9 | U2 |
|  | SM(d18:016:1(9Z)) | 703.5746 | M+H | 1495.3 | U2 |
|  | 3-O-sulfogalactosylceramide (d18:122:0) | 864.6247 | M+H | 1107.2 | U2 |
|  | 3-O-sulfogalactosylceramide (d18:116:0) | 401.7604  780.5301 | M+H+Na  M+H | 848.8 | U2 |
|  | 3-O-sulfogalactosylceramide (d18:120:0) | 429.7918  836.5937  418.8004 | M+H+Na  M+H  M+2H | 1039.7 | U2 |
|  | DHAP(10:0) | 325.1415 | M+H | 1111 | U2 |
|  | LPA(P-16:0e0:0) | 395.2543 | M+H | 821.5 | U2 |
|  | TG(22:2/20:2/20:5)/TG(22:2/18:2/20:5)/TG(20:3/22:4/20:2)/  TG(20:3/22:4/18:2)/TG(20:3/20:2/22:4)/TG(20:3/18:2/22:4)/  TG(20:2/22:4/20:3)/TG(20:2/22:2/20:5)/TG(20:2/20:5/22:2)/  TG(20:2/20:3/22:4)/TG(20:2/20:1/22:6)/TG(20:1/22:6/20:2)/  TG(20:1/22:6/18:2)/TG(20:1/20:2/22:6)/TG(20:1/18:2/22:6)/  TG(18:2/22:4/20:3)/TG(18:2/22:2/20:5)/TG(18:2/20:5/22:2)/  TG(18:2/20:3/22:4)/TG(18:2/20:1/22:6) | 971.8046  990.7874 | M+H  2M+H+K | 118.8 | M3 |
| STEROID HORMONES | 17-beta-estradiol 3-sulfate-17-(beta-D-glucuronide) | 529.1743 | M+H | 728.7 | U2 |
|  | tetrahydroaldosterone-3-glucuronide | 541.2642 | M+H | 805.6 | U2 |
|  | 11-beta-hydroxyandrosterone-3-glucuronide | 483.2565 | M+H | 1088.4 | U2 |
|  | 6-dehydrotestosterone glucuronide | 463.2324 | M+H | 1050.1 | U2 |
|  | 17a,21-dihydroxy-5b-pregnane-3,11,20-trione/cortisol/  18-hydroxycorticosterone | 364.2198  363.2166 | M+H_[+1]  M+H | 850.3 | M3 |
|  | 21-hydroxy-5b-pregnane-3,11,20-trione/ 21-deoxycortisol/  corticosterone/cortexolone/19-hydroxydeoxycorticosterone | 348.2253  347.2217 | M+H_[+1]  M+H | 805.9 | M3 |
|  | 3-alpha-hydroxy-5-alpha-androstane-17-one 3-d-glucuronide/  5-alpha-dihydrotestosterone glucuronide/androsterone glucuronide | 468.2648  467.2619 | M+H_[+1]  M+H | 1151.8 | M3 |
|  | 16-alpha17-beta-estriol 17-beta-d-glucuronide/estriol-3-glucuronide/  estriol-16-glucuronide/15-hydroxynorandrostene-317-dione glucuronide/  estriol-17-glucuronide | 466.2143  465.2113 | M+H_[+1]  M+H | 813.8 | M3 |
|  | 17-alpha-estradiol-3-glucuronide/17-beta-estradiol glucuronide/  17-beta-estradiol-3-glucuronide/2-methoxyestrone 3-glucuronide/  estradiol-17alpha 3-D-glucuronoside | 450.2201  449.2166 | M+H_[+1]  M+H | 903.1 | M3 |
|  | cortolone-3-glucuronide | 565.2622  525.2696  544.2835  543.2801 | M+Na  M+H-H2O  M+H_[+1]  M+H | 798.6 | U3 |
|  |  | 542.2695  541.266 | M-H_[-1]  M-H | 724 | U3 |
|  | methyltestosterone/eicosapentaenoic acid | 302.2209  301.2176 | M-H_[-1]  M-H | 1359.1 | M3 |
|  | 5a-dihydrotestosterone sulfate/androsterone sulfate | 370.1776  369.1742 | M-H_[-1]  M-H | 895.5 | M3 |
|  | tetrahydroaldosterone-3-glucuronide | 539.2503 | M-H | 751.1 | U2 |
|  | 11-beta-hydroxyandrosterone-3-glucuronide | 481.2446 | M-H | 765.2 | U2 |
| LEUKOTRIENES | 20-COOH-leukotriene E4 | 468.2084 | M-H | 968.7 | U2 |
|  | leukotriene E4 | 440.2486 | M+H | 898 | U2 |
| ACYLCARNITINES | L-acetylcarnitine | 205,12668  204,12333 | M+H_[+1]  M+H | 646.3 | U3 |
|  |  | 202.1079 | M-H | 90.6 | U2 |
|  | L-carnitine | 163,11598  162,11266 | M+H_[+1]  M+H | 234 | U2 |
|  | propionylcarnitine | 219,14268  218,13872 | M+H  M+H | 811.8 | U3 |
|  | 3-dehydroxycarnitine | 146.1178 | M+H | 402.3 | U2 |
| PURINES,  PYRIMIDINES | N4-acetylcytidine | 286,10346  308,08534  324.0593  287.1068 | M+H  M+Na  M+K  M+H_[+1] | 509.3 | U3 |
|  | 5-methylthioadenosine | 298,09685 | M+H | 746.6 | U2 |
|  | 1-methylinosine | 284.1072  283.1038  305.0857  321.0597 | M+H_[+1]  M+H  2M+2Na  2M+2K | 498.4 | U2 |
|  | 7-methylguanosine | 299.1239 | M+H | 467.8 | U2 |
|  | S-adenosylhomocysteine | 385,12883 | M+H | 604.1 | U2 |
|  | CDP | 404.0263 | M+H | 691.1 | U2 |
|  | N6-carbamoyl-L-threonyladenosine | 413.1416 | M+H | 590.7 | U2 |
| AMINO ACIDS AND RELATED METABOLITES | L-leucine/L-isoleucine | 133,1055  132,10221 | M+H_[+1]  M+H | 493.4 | M3 |
|  | N6,N6,N6-trimethyl-L-lysine | 189,16005 | M+H | 236 | U2 |
|  | galactosylhydroxylysine | 326,16349  325,16024 | M+H_[+1]  M+H | 1032.7 | U2 |
|  | L-glutamic acid | 148,06064 | M+H | 99.6 | U2 |
|  | L-phenylalanine | 169,09341  167,08951  166,08651  207,11305 | M+H_[+3]  M+H_[+1]  M+H  M+ACN+H | 653.3 | M3 |
|  | L-methionine | 152,05436  151,06188  150,05859 | M+H_[+2]  M+H_[+1]  M+H | 246.8 | U2 |
|  | ornithine | 134,10078  115,08704  133.09744 | M+H_[+1]  M+H-H2O  M+H | 131.2 | U2 |
|  | L-histidine | 156.07705 | M+H | 140.7 | U2 |
|  | L-tyrosine | 184,08831  183,08456  182,08141  223,1077  226,04515 | M+H_[+2]  M+H_[+1]  M+H  M+ACN+H  M+2Na-H | 527 | U2 |
|  | L-proline | 117.0744  116.071 | M+H_[+1]  M+H | 107.4 | U3 |
|  | creatine | 154.0589  132.077 | M+Na  M+H | 152.4 | U3 |
|  | indoleacetyl glutamine | 304.1293 | M+H | 689 | U2 |
|  | L-arginine | 175.1192  176,11662 | M+H  M+H_[+1] | 172.5 | U2 |
|  | L-threonine | 120.0659 | M+H | 94.5 | U2 |
|  | betaine | 118.0867 | M+H | 92.8 | U2 |
|  | 2-phenylglycine | 174.0528  152.0709  325.1159 | M+Na  M+H  2M+Na | 546.3 | M3 |
|  | indoleacetyl glutamine | 302.1149 | M-H | 648.3 | U2 |
|  | L-cystine | 239.0163 | M-H | 69.7 | U2 |
| PEPTIDES | dynorphin B (10-13) | 446,296 | M+H | 667.9 | U2 |
|  | L-cysteinylglycine disulfide | 298.0526 | M+H | 132 | U2 |
|  | N-acetylaspartylglutamic acid | 325.0655  304.087  303.0835 | M+Na-2H  M-H_[-1]  M-H | 141.1 | U3 |
|  | tryptophyl-lysine/lysyl-tryptophan | 332.1798  331.1764 | M-H_[-1]  M-H | 862 | M3 |
|  | glycylprolylhydroxyproline | 286.1399 | M+H | 511.9 | U2 |
| OTHER | creatinine | 115.0699  114.0666  249.10721  136.0484  227.12523 | M+H_[+1]  M+H  2M+Na  M+Na  2M+H | 225.4 | U3 |
|  | urothion | 326.0377 | M+H | 608.9 | U2 |
|  | L-urobilinogen | 597.36263 | M+H | 891.7 | U2 |
|  | cholesterol sulfate | 467.32  489.3021 | M+H  2M+2Na | 911.6 | U3 |
|  | dopamine quinone | 174.0528  152.0709  325.1159 | M+Na  M+H  2M+Na | 546.3 | M3 |
|  | norsalsolinol | 167.0895  166.0865  207.1131 | M+H_[+1]  M+H  M+ACN+H | 651.2 | M3 |

**Table 6** The list of annotated compounds (metabolites) in each analyzed group of patients and healthy controls. Only unique and multiple features with medium to high confidence level were selected and annotated by HMDB. The annotation was based on intensity profiles, retention time, mass defect, and isotope/adduct patterns of peaks. In high confidence match, non-zero xMSannotator multistage score, required adducts, N, O, P, S/C ratio check, hydrogen/carbon ratio check, abundance ratio checks for isotopes, multimers and multiply charged adducts are satisfied. In medium confidence match, pathway level correlation is satisfied.

|  | **CONTROL** | **CONVERTER_ BASELINE** | **CONVERTER_ FOLLOWUP** | **NONCONVERTER_ BASELINE** | **NONCONVERTER_ FOLLOWUP** | **PsA Mild** | **PsA Moderate** | **PsA Severe** |
| --- | --- | --- | --- | --- | --- | --- | --- | --- |
|  |  |  |  |  |  |  |  |  |
| **I. amino acids,** | Beta-Leucine | 5-Oxoprolinate (Pyroglutamic acid) | L-Leucine | N-Acetyl-S-(N-methylcarbamoyl)cysteine | L-Tryptophan | L-Leucine | L-Tryptophan | Galactosylhydroxylysine |
| **their precursors** | D-Proline | D-Arginine | 5-Hydroxy-L-tryptophan | L-Tryptophan | L-Pipecolic acid | L-Isoleucine | L-Methionine | Ornithine |
| **and metabolites** | L-Norleucine | L-Valine | L-Isoleucine | L-Tyrosine | L-Leucine | L-Phenylalanine | L-Phenylalanine | 5-Oxoprolinate |
|  | L-Tryptophan | L-Methionine | N6,N6,N6-Trimethyl-L-lysine | N-Alpha-acetyllysine | 3-Methylhistidine | Thiocysteine | D-Proline | L-Allothreonine |
|  | L-Leucine | L-Leucine | L-Methionine | N6-Acetyl-L-lysine | L-Isoleucine | Cysteic acid | D-Ornithine | L-Homoserine |
|  | L-Alloisoleucine | Homocitrulline | Ornithine | L-Tryptophan | L-Proline | N6,N6,N6-Trimethyl-L-lysine | L-Leucine | Pyroglutamic acid |
|  | L-Isoleucine | L-Arginine | L-Cystine | D-Serine | L-Tyrosine | L-Arginine | L-Isoleucine | L-Threonine |
|  | L-Proline | Pyroglutamic acid | L-Lysine | Cysteic acid | Pipecolic acid | Ornithine | L-Proline | L-Phenylalanine |
|  | L-Lysine | L-Isoleucine | L-Asparagine | Thiocysteine | 1-Methylhistidine | L-Histidine | Thiocysteine | L-Tryptophan |
|  | L-Histidine | Thiocysteine | L-Phenylalanine | Tridecanoylglycine | Ne,Ne dimethyllysine | L-Threonine | N6,N6,N6-Trimethyl-L-lysine | L-Glutamine |
|  | N6,N6,N6-Trimethyl-L-lysine | D-Serine | L-Glutamic acid | N-Undecanoylglycine | Thiocysteine | N-Formylkynurenine | L-Histidine | L-Arginine |
|  | N-Formylkynurenine | N6,N6,N6-Trimethyl-L-lysine | Thiocysteine | N-Nonanoylglycine | N6,N6,N6-Trimethyl-L-lysine | Kynurenic acid | Kynurenic acid | L-Histidine |
|  | L-Kynurenine | Ornithine | Tridecanoylglycine | N-Lauroylglycine | L-Phenylalanine | Tridecanoylglycine | Tridecanoylglycine | L-Proline |
|  | Kynurenic acid | L-Lysine | N-Undecanoylglycine | N-Decanoylglycine | Ornithine | N-Undecanoylglycine | N-Undecanoylglycine | L-Tyrosine |
|  | Tridecanoylglycine | L-Histidine | N-Nonanoylglycine | N-Heptanoylglycine | L-Kynurenine | N-Nonanoylglycine | N-Nonanoylglycine | L-Glutamic acid |
|  | N-Undecanoylglycine | L-Threonine | N-Lauroylglycine | Capryloylglycine | Tridecanoylglycine | N-Nonanoylglycine | N-Lauroylglycine | Tridecanoylglycine |
|  | N-Nonanoylglycine | L-Tyrosine | N-Decanoylglycine |  | N-Nonanoylglycine | N-Lauroylglycine | N-Decanoylglycine | N-Undecanoylglycine |
|  | N-Lauroylglycine | L-Glutamic acid | N-Heptanoylglycine |  | N-Lauroylglycine |  |  | N-Nonanoylglycine |
|  | N-Decanoylglycine | L-Kynurenine |  |  | N-Nonanoylglycine |  |  | N-Lauroylglycine |
|  |  | Kynurenic acid |  |  | N-Undecanoylglycine |  |  | N-Decanoylglycine |
|  |  | Tridecanoylglycine |  |  | N-Decanoylglycine |  |  | N-Heptanoylglycine |
|  |  | N-Undecanoylglycine |  |  | N-Heptanoylglycine |  |  | N-Acryloylglycine |
|  |  | N-Nonanoylglycine |  |  | Capryloylglycine |  |  |  |
|  |  | N-Lauroylglycine |  |  |  |  |  |  |
|  |  | N-Decanoylglycine |  |  |  |  |  |  |
|  |  | Tiglylglycine |  |  |  |  |  |  |
|  |  | N-Acryloylglycine |  |  |  |  |  |  |
| **II. peptides** | Kinetensin 1-3 | Kinetensin 1-3 | Kinetensin 1-3 | Kinetensin 1-3 | Tryptophyl-Glycine | Kinetensin 1-3 | Kinetensin 1-3 | Kinetensin 1-3 |
|  | Histidinyl-Glycine | Tyrosyl-Glycine | Tyrosyl-Hydroxyproline | Valyl-Alanine | Methionyl-Arginine | Tyrosyl-Glycine | Tryptophyl-Arginine | Tyrosyl-Glycine |
|  | Glycyl-Histidine | Phenylalanyl-Glycine | Leucyl-Proline | Lysyl-Glutamate | Leucyl-Alanine | Phenylalanyl-Glycine | Leucyl-Proline | Glycyl-Tyrosine |
|  | Arginyl-Glycine | Lysyl-Glutamate | Isoleucyl-Proline | Leucyl-Glycine | Isoleucyl-Alanine | Methionyl-Arginine | Isoleucyl-Proline | Gamma-Glutamyltyrosine |
|  | Gamma-Glutamyltyrosine | Glycyl-Tyrosine | Hydroxyprolyl-Tyrosine | Isoleucyl-Glycine | Glycyl-Tryptophan | Histidinyl-Hydroxyproline | Histidinyl-Glycine | N-Acetylaspartylglutamic acid |
|  | N-Acetylaspartylglutamic acid | Glycyl-Phenylalanine | Asparaginyl-Arginine | Histidinyl-Glycine | Arginyl-Methionine | Hydroxyprolyl-Histidine | Glycyl-Histidine |  |
|  |  | Glutamyl-Lysine | Arginyl-Asparagine | Glycyl-Isoleucine | Alanyl-Leucine | Glycyl-Tyrosine | Glycyl-Arginine |  |
|  |  | Asparaginyl-Arginine | Glutamylphenylalanine | Glycyl-Histidine | Alanyl-Isoleucine | Glycyl-Phenylalanine | Arginyl-Tryptophan |  |
|  |  | Arginyl-Asparagine | N-gamma-L-Glutamyl-L-phenylalanine | Glutamyl-Lysine | Gamma-Glutamyltyrosine | Asparaginyl-Arginine | Arginyl-Glycine |  |
|  |  | Gamma-Glutamyltyrosine | L-leucyl-L-proline | Asparaginyl-Arginine | N-Acetylaspartylglutamic acid | Arginyl-Methionine | L-leucyl-L-proline |  |
|  |  |  | L-isoleucyl-L-proline | Arginyl-Asparagine |  | Arginyl-Asparagine | L-isoleucyl-L-proline |  |
|  |  |  | Gamma-Glutamyltyrosine | Alanyl-Valine |  | gamma-Glutamyl-S-methylcysteinyl-beta-alanine | N-gamma-L-Glutamyl-L-methionine |  |
|  |  |  | N-Acetylaspartylglutamic acid | Glycyl-L-leucine |  | N-gamma-L-Glutamyl-L-methionine | Gamma-Glutamyltyrosine |  |
|  |  |  |  | Gamma-Glutamyltyrosine |  | N-Acetylaspartylglutamic acid | D-Pantothenoyl-L-cysteine |  |
|  |  |  |  |  |  | Gamma-Glutamyltyrosine | N-Acetylaspartylglutamic acid |  |
| **III. acylcarnitines** | 3-Hydroxy-9-hexadecenoylcarnitine | Propionylcarnitine | Propionylcarnitine | 3-Hydroxyhexadecadienoylcarnitine | 6-Keto-decanoylcarnitine | 3-Hydroxy-9-hexadecenoylcarnitine | 3-Hydroxyhexadecadienoylcarnitine | 3-Hydroxyhexadecadienoylcarnitine |
|  | 2-trans4-cis-Decadienoylcarnitine | 3-Hydroxyhexadecadienoylcarnitine | 3-Hydroxyhexadecadienoylcarnitine | Heptanoylcarnitine | 2-Hexenoylcarnitine | Heptanoylcarnitine | 3-Hydroxy-9-hexadecenoylcarnitine | Heptanoylcarnitine |
|  | Heptanoylcarnitine | Heptanoylcarnitine | 3-Hydroxy-9-hexadecenoylcarnitine | 6-Keto-decanoylcarnitine | 3-Dehydroxycarnitine | 6-Keto-decanoylcarnitine | Heptanoylcarnitine | 6-Keto-decanoylcarnitine |
|  | 6-Keto-decanoylcarnitine | 9-Decenoylcarnitine | 2-trans4-cis-Decadienoylcarnitine | Hydroxybutyrylcarnitine | Propionylcarnitine | 2-Hexenoylcarnitine | Hydroxybutyrylcarnitine | Hydroxybutyrylcarnitine |
|  | 2-Hexenoylcarnitine | Hydroxybutyrylcarnitine | 9-Decenoylcarnitine | 3-Dehydroxycarnitine | L-Acetylcarnitine | Hydroxybutyrylcarnitine | 3-Dehydroxycarnitine | 3-Dehydroxycarnitine |
|  | Hydroxybutyrylcarnitine | 3-Dehydroxycarnitine | 6-Keto-decanoylcarnitine | Propionylcarnitine | L-Carnitine | 3-Dehydroxycarnitine | Propionylcarnitine | Propionylcarnitine |
|  | 3-Dehydroxycarnitine | L-Acetylcarnitine | Hydroxybutyrylcarnitine | 3-Methylglutarylcarnitine |  | Propionylcarnitine | 3-Methylglutarylcarnitine | Tiglylcarnitine |
|  | Propionylcarnitine | L-Carnitine | 3-Dehydroxycarnitine | L-Acetylcarnitine |  | 3-Methylglutarylcarnitine | L-Acetylcarnitine | L-Acetylcarnitine |
|  | 3-Methylglutarylcarnitine |  | 3-Methylglutarylcarnitine | L-Carnitine |  | L-Octanoylcarnitine | L-Carnitine |  |
|  | L-Octanoylcarnitine |  | L-Acetylcarnitine |  |  | L-Acetylcarnitine |  |  |
|  | L-Acetylcarnitine |  | L-Carnitine |  |  | L-Carnitine |  |  |
|  | L-Carnitine |  |  |  |  |  |  |  |
| **IV. Eisocanoids** |  |  |  |  |  |  |  |  |
| **1. prostaglandins** | 5,6-Dihydroxyprostaglandin F1a | 5,6-Dihydroxyprostaglandin F1a | 5,6-Dihydroxyprostaglandin F1a | 5,6-Dihydroxyprostaglandin F1a | 5,6-Dihydroxyprostaglandin F1a | 5,6-Dihydroxyprostaglandin F1a | 5,6-Dihydroxyprostaglandin F1a | 5,6-Dihydroxyprostaglandin F1a |
|  |  |  | 13,14-Dihydro PGF2a |  |  |  | Prostaglandin C1 | Prostaglandin C1 |
|  |  |  | 13,14-Dihydro PGE1 |  |  |  | 9-Deoxy-delta12-PGD2 | 9-Deoxy-delta12-PGD2 |
|  |  |  | Prostaglandin F1a |  |  |  | Prostaglandin B1 | Prostaglandin B1 |
|  |  |  |  |  |  |  | Prostaglandin A1 | Prostaglandin A1 |
|  |  |  |  |  |  |  | 8-iso-PGA1 | 8-iso-PGA1 |
| **2. leukotrienes** | Omega-Carboxy-trinor-leukotriene B4 | Omega-Carboxy-trinor-leukotriene B4 | Omega-Carboxy-trinor-leukotriene B4 | Omega-Carboxy-trinor-leukotriene B4 | 1011-dihydro-20-trihydroxy-leukotriene B4 | Omega-Carboxy-trinor-leukotriene B4 | Omega-Carboxy-trinor-leukotriene B4 | Omega-Carboxy-trinor-leukotriene B4 |
|  | 10,11-dihydro-20-trihydroxy-leukotriene B4 | 10,11-dihydro-20-trihydroxy-leukotriene B4 | 10,11-dihydro-20-trihydroxy-leukotriene B4 | 10,11-dihydro-20-trihydroxy-leukotriene B4 |  | 10,11-Dihydro-12R-hydroxy-leukotriene E4 | 10,11-dihydro-20-trihydroxy-leukotriene B4 | 10,11-dihydro-20-trihydroxy-leukotriene B4 |
|  | 10,11-dihydro-12R-hydroxy-leukotriene E4 |  | 10,11-Dihydro-12R-hydroxy-leukotriene E4 | 10,11-Dihydro-12R-hydroxy-leukotriene E4 |  | 10,11-dihydro-20-trihydroxy-leukotriene B4 | 10,11-Dihydro-12R-hydroxy-leukotriene E4 | 10,11-Dihydro-12R-hydroxy-leukotriene E4 |
|  |  |  | Leukotriene C5 |  |  |  | Leukotriene C5 | Leukotriene C5 |
|  |  |  |  |  |  |  | Leukotriene B4 | Leukotriene B4 |
|  |  |  |  |  |  |  | 6,7-dihydro-5-oxo-12-epi-LTB4 | 6,7-dihydro-5-oxo-12-epi-LTB4 |
|  |  |  |  |  |  |  | 10,11-dihydro-12-oxo-LTB4 | 10,11-dihydro-12-oxo-LTB4 |
|  |  |  |  |  |  |  | 12(S)-Leukotriene B4 | 12(S)-Leukotriene B4 |
|  |  |  |  |  |  |  | 6-trans-12-epi-Leukotriene B4 | 6-trans-12-epi-Leukotriene B4 |
|  |  |  |  |  |  |  | 6-trans-Leukotriene B4 | 6-trans-Leukotriene B4 |
| **3. other** |  | 9-HODE | 9-HODE | 9-HODE |  | 9-HODE | 9-HODE | 9-HODE |
|  |  | 12,13-EpOME | 12,13-EpOME | 12,13-EpOME |  | 12,13-EpOME | 12,13-EpOME | 12,13-EpOME |
|  |  |  | 9,10-DHOME |  |  |  | 12,20-DiHETE | 12,20-DiHETE |
|  |  |  | 12,13-DHOME |  |  |  | 8,15-DiHETE | 8,15-DiHETE |
|  |  |  |  |  |  |  | 5,15-DiHETE | 5,15-DiHETE |
|  |  |  |  |  |  |  | 17,18-DiHETE | 17,18-DiHETE |
|  |  |  |  |  |  |  | 14,15-DiHETE | 14,15-DiHETE |
|  |  |  |  |  |  |  | 11H-14,15-EETA | 11H-14,15-EETA |
|  |  |  |  |  |  |  | 15H-11,12-EETA | 15H-11,12-EETA |
|  |  |  |  |  |  |  | 5-HPETE | 5-HPETE |
|  |  |  |  |  |  |  | 8(S)-HPETE | 8(S)-HPETE |
|  |  |  |  |  |  |  | 11(R)-HPETE | 11(R)-HPETE |
|  |  |  |  |  |  |  | 12(R)-HPETE | 12(R)-HPETE |
|  |  |  |  |  |  |  | 15(S)-HPETE | 15(S)-HPETE |
|  |  |  |  |  |  |  | 12(S)-HPETE | 12(S)-HPETE |
|  |  |  |  |  |  |  |  |  |
|  |  |  |  |  |  |  |  |  |
|  |  |  |  |  |  |  |  |  |
| **V. lipids** | LysoPC(20:5) | LysoPC(22:6) | LysoPC(20:5) | LysoPC(14:0) | LysoPC(22:6) | LysoPC(22:6) | LysoPC(20:5) | LysoPC(18:2) |
|  | LysoPC(18:2) | LysoPC(20:5) | LysoPC(16:0) | LysoPC(20:4) | PG(18:2/16:0) | LysoPC(20:5) | LysoPC(18:2) | LysoPC(16:0) |
|  | LysoPC(14:0) | LysoPC(16:1(9) | LysoPC(0:018:0) | LysoPC(20:4) | PG(18:1/16:1) | LysoPC(18:4) | LysoPC(16:0) | LysoPC(18:1) |
|  | LysoPC(0:018:0) | LysoPC(16:0) | LysoPC(18:0) | LysoPC(18:1) | PG(18:1/16:1) | LysoPC(18:2) | LysoPC(0:018:0) | LysoPC(18:1) |
|  | LysoPC(18:2) | LysoPC(0:018:0) | LysoPC(16:0) | LysoPC(18:1) | PG(16:1/18:1) | LysoPC(16:1) | LysoPC(22:6) | LysoPC(22:6) |
|  | LysoPC(18:0) | LysoPC(18:1) | LysoPC(22:6) | LysoPC(22:6) | PG(16:1/18:1) | LysoPC(16:0) | LysoPC(18:4) | LysoPC(18:4) |
|  | LysoPC(22:6) | LysoPC(18:0) | LysoPC(18:4) | LysoPC(20:5) | PG(16:018:2) | PS(18:018:0) | LysoPC(20:4) | LysoPC(16:1) |
|  | LysoPC(18:4) | LysoPC(18:1) | LysoPC(18:2) | LysoPC(18:4) | SM(d18:016:1) | PI(16:016:0) | LysoPC(20:4) | TG(22:2/20:2/20:5) |
|  | LysoPC(16:1) | LysoPC(18:4) | LysoPC(16:1) | LysoPC(18:2) | PE(24:024:0) | PE(24:024:0) | LysoPC(18:3) | TG(22:2/18:2/20:5) |
|  | LysoPC(16:0) | LysoPC(18:2) | PI(16:016:0) | LysoPC(16:1) | MG(15:00:00:0) | PE(14:014:0) | LysoPC(18:3) | TG(20:3/20:2/22:4) |
|  | LysoPE(22:6/0:0) | PI(16:016:0) | PE(24:024:0) | LysoPC(16:0) | MG(0:015:00:0) | Oleamide | LysoPC(18:2) | TG(20:3/18:2/22:4) |
|  | LysoPE(0:022:6) | PE(24:024:0) | PE(14:014:0) | LysoPE(20:4/0:0) | Arachidic acid | Docosanamide | LysoPC(18:0) | TG(20:2/20:5/22:2) |
|  | PI(16:016:0) | PE(14:014:0) | MG(15:00:00:0) | LysoPE(20:4/0:0) | Undecanedioic acid | 5Z-Tetradecenoic acid | LysoPC(16:1) | TG(20:2/22:4/20:3) |
|  | PE(24:024:0) | MG(15:00:00:0) | MG(14:00:00:0) | LysoPE(20:3/0:0) | 5-Dodecenoic acid | 5-Tetradecenoic acid | PE(24:024:0) | TG(20:2/22:2/20:5) |
|  | PE(14:014:0) | MG(0:015:00:0) | MG(0:015:00:0) | LysoPE(20:3/0:0) | Sphinganine 1-phosphate | Palmitic amide | PE(14:014:0) | TG(20:2/20:3/22:4) |
|  | MG(16:00:00:0) | 5-Dodecenoic acid | MG(0:014:00:0) | LysoPE(20:3/0:0) |  | 910-Epoxyoctadecenoic acid | MG(14:00:00:0) | TG(20:2/20:3/22:4) |
|  | MG(15:00:00:0) | Sphinganine 1-phosphate | Tetradecanedioic acid | LysoPE(0:020:4) |  | 13S-hydroxyoctadecadienoic acid | MG(0:014:00:0) | TG(20:2/20:1/22:6) |
|  | MG(0:016:00:0) | Palmitic amide | 3-O-Sulfogalactosylceramide (d18:122:0) | LysoPE(0:020:4) |  |  | Oleamide | TG(18:2/20:5/22:2) |
|  | MG(0:015:00:0) | Oleamide | Palmitic amide | LysoPE(0:020:3) |  |  | Docosanamide | TG(18:2/22:4/20:3) |
|  | Dodecanedioic acid | Tetracosahexaenoic acid n-3 | Oleamide | LysoPE(0:020:3) |  |  | Arachidic acid | TG(18:2/22:2/20:5) |
|  | 5-Dodecenoic acid | Docosanamide | Docosanamide | LysoPE(0:020:3) |  |  | Undecanedioic acid | TG(18:2/20:3/22:4) |
|  | Palmitic amide |  |  | PI(16:016:0) |  |  | Palmitic amide | TG(18:2/20:3/22:4) |
|  | Oleamide |  |  | PE(24:024:0) |  |  | 3-Oxooctadecanoic acid | TG(18:2/20:1/22:6) |
|  | Octadecanamide |  |  | PE(14:014:0) |  |  | 910-Epoxyoctadecenoic acid | TG(20:3/22:4/20:2) |
|  | Docosanamide |  |  | Oleamide |  |  | 13S-hydroxyoctadecadienoic acid | TG(20:3/22:4/18:2) |
|  |  |  |  | Docosanamide |  |  |  | TG(20:3/20:2/22:4) |
|  |  |  |  | Palmitic amide |  |  |  | TG(20:3/18:2/22:4) |
|  |  |  |  | 3-Oxohexadecanoic acid |  |  |  | TG(20:1/22:6/20:2) |
|  |  |  |  | 9,10-Epoxyoctadecenoic acid |  |  |  | TG(20:1/22:6/18:2) |
|  |  |  |  |  |  |  |  | TG(20:1/20:2/22:6) |
|  |  |  |  |  |  |  |  | TG(20:1/18:2/22:6) |
|  |  |  |  |  |  |  |  | PS(18:018:0) |
|  |  |  |  |  |  |  |  | PS(18:020:0) |
|  |  |  |  |  |  |  |  | PI(16:016:0) |
|  |  |  |  |  |  |  |  | PE(24:024:0) |
|  |  |  |  |  |  |  |  | PE(14:014:0) |
|  |  |  |  |  |  |  |  | MG(16:00:00:0) |
|  |  |  |  |  |  |  |  | MG(0:016:00:0) |
|  |  |  |  |  |  |  |  | Oleamide |
|  |  |  |  |  |  |  |  | Docosanamide |
|  |  |  |  |  |  |  |  | Palmitic amide |
| **VI. hormones** |  | 17a,21-Dihydroxy-5b-pregnane-3,11,20-trione | 17a,21-Dihydroxy-5b-pregnane-3,11,20-trione |  | 19-Oic-deoxycorticosterone |  |  | Tetrahydroaldosterone-3-glucuronide |
|  |  | Cortisol | Cortisol |  | Cortisone |  |  | Pregnenolone sulfate |
|  |  | Tetrahydroaldosterone-3-glucuronide | Tetrahydroaldosterone-3-glucuronide |  | Aldosterone |  |  |  |
|  |  |  | 3b,16a-Dihydroxyandrostenone sulfate |  |  |  |  |  |
|  |  |  | 18-Hydroxycorticosterone |  |  |  |  |  |
| **VII. purines, pyrimidines** | N4-Acetylcytidine | N4-Acetylcytidine | N4-Acetylcytidine | 5-Amino-6-ribitylamino uracil | dIMP | N4-Acetylcytidine | N4-Acetylcytidine | N4-Acetylcytidine |
|  | 1-Methyladenosine | 1-Methyladenosine | dCMP | 5-Acetylamino-6-formylamino-3-methyluracil | N4-Acetylcytidine | S-Adenosylmethioninamine | N2-Methylguanine | di-Hydroxymelatonin |
|  | 3-O-Methyladenosine | 3-O-Methyladenosine | 1-Methylinosine | N4-Acetylcytidine | 3-O-Methyladenosine | S-Adenosylhomocysteine | 7-Methylguanine | N2,N2-Dimethylguanosine |
|  | 2-O-Methyladenosine | 2-O-Methyladenosine | Cytidine 2,3-cyclic phosphate | S-Adenosylmethioninamine | N2N2-Dimethylguanosine | Cytidine monophosphate | 1-Methylguanine | 17-Dimethylguanosine |
|  | N6-Methyladenosine | N6-Methyladenosine | 5-Amino-6-ribitylamino uracil | Cytidine monophosphate | 2-O-Methyladenosine | dIMP | Cytidine 2,3-cyclic phosphate | Cytidine 2,3-cyclic phosphate |
|  | 5-Amino-6-ribitylamino uracil | 1-Methylguanine | 5-Acetylamino-6-formylamino-3-methyluracil |  | N6-Methyladenosine |  | 5-Amino-6-ribitylamino uracil | 5-Amino-6-ribitylamino uracil |
|  | 5-Acetylamino-6-formylamino-3-methyluracil | 3-Methylguanine | Cysteic acid |  | 1-Methyladenosine |  | 5-Acetylamino-6-formylamino-3-methyluracil | 5-Acetylamino-6-formylamino-3-methyluracil |
|  | S-Adenosylmethioninamine | 7-Methylguanine | 5-Methylthioadenosine |  | 17-Dimethylguanosine |  | 5-Methylthioadenosine | 5-Methylthioadenosine |
|  | 5-Methylthioadenosine | Cytidine 2,3-cyclic phosphate | S-Adenosylmethioninamine |  | Cytidine 23-cyclic phosphate |  | S-Adenosylmethioninamine | S-Adenosylmethioninamine |
|  | 1-Methylinosine | 5-Amino-6-ribitylamino uracil | Cytosine |  | 5-Amino-6-ribitylamino uracil |  |  | dIMP |
|  |  | 5-Methylthioadenosine | Cytidine monophosphate |  | 5-Acetylamino-6-formylamino-3-methyluracil |  |  |  |
|  |  | S-Adenosylmethioninamine |  |  | N4-Acetylcytidine |  |  |  |
|  |  | S-Adenosylhomocysteine |  |  | 5-Methylthioadenosine |  |  |  |
|  |  | N2-Methylguanine |  |  | S-Adenosylmethioninamine |  |  |  |
|  |  |  |  |  | S-Adenosylhomocysteine |  |  |  |
| **VIII. other meatbolites** | Creatinine | Creatine | Creatinine | Creatinine | L-Methionine | Dynorphin A (6-8) | Alpha-D-Glucose | Ubiquinone-1 |
|  | Phosphohydroxypyruvic acid | Formyl-5-hydroxykynurenamine | Creatine | di-Hydroxymelatonin | L-Tyrosine | Beta-Carboline | Creatinine | Phosphohydroxypyruvic acid |
|  | Alpha-D-Glucose | Betaine | Protoporphyrinogen IX | Protoporphyrinogen IX | 4-O-Methyl-myo-inositol | Norepinephrine sulfate | 5-Hydroxyindoleacetic acid | Dynorphin B (10-13) |
|  | L-Methionine | Dynorphin B (10-13) | N-Acetyl-D-glucosamine | 5-Hydroxyindoleacetic acid | D-4-O-Methyl-myo-inositol | L-Methionine | Creatine | Dynorphin A (6-8) |
|  | Cholesterol sulfate | Alpha-D-Glucose | N-gamma-L-Glutamyl-L-methionine | Dynorphin A (6-8) | 5-Hydroxyindoleacetaldehyde | Porphobilinogen | Dynorphin A (6-8) | Alpha-D-Glucose |
|  | Norepinephrine sulfate | Norepinephrine sulfate | Dynorphin A (6-8) | Galactosylhydroxylysine | Alpha-D-Glucose | Betaine | Urothion | Guanidinosuccinic acid |
|  | Porphobilinogen | Creatinine | Norepinephrine sulfate | Alpha-D-Glucose | Protoporphyrinogen IX | Alpha-D-Glucose | Norepinephrine sulfate | Urothion |
|  | Urothion (in urine) | Porphobilinogen | Taurine | Urothion | 5-Hydroxyindoleacetic acid | Creatinine | 78-Dihydropteroic acid | Cholesterol glucuronide |
|  | Farnesylcysteine | Farnesylcysteine | Porphobilinogen | Norepinephrine sulfate | Phosphocreatinine | Urothion | Cholesterol sulfate | Norepinephrine sulfate |
|  |  | Enkephalin L | Urothion | Porphobilinogen | Kinetensin 1-3 | Farnesylcysteine | Porphobilinogen | Cholesterol sulfate |
|  |  |  |  | Farnesylcysteine | Dynorphin B (10-13) |  | Farnesylcysteine | Creatinine |
|  |  |  |  |  | L-Urobilinogen |  |  | Taurine |
|  |  |  |  |  | Guanidinosuccinic acid |  |  | Porphobilinogen |
|  |  |  |  |  | Norepinephrine sulfate |  |  | di-Hydroxymelatonin |
|  |  |  |  |  | Creatinine |  |  |  |
|  |  |  |  |  | Farnesylcysteine |  |  |  |
|  |  |  |  |  |  |  |  |  |
